# A Three-Layer Adaptive Kalman Filter Approach for Multi-Source Time Fusion Using Temperature-Compensated Oscillators with GNSS and eLoran Backup

**DOI:** 10.3390/s26175397

**Published:** 2026-08-26

**Authors:** Ziming Yuan, Shuaihe Gao, Pengfei Li, Shougang Zhang

**Affiliations:** 1 National Time Service Center, Chinese Academy of Sciences, Xi’an 710600, China; yuanziming24@mails.ucas.ac.cn (Z.Y.); zhangshougang@ntsc.ac.cn (S.Z.); 2 University of Chinese Academy of Sciences, Beijing 100039, China; 3 School of Measurement-Control Technology and Communications Engineering, Harbin University of Science and Technology, Harbin 150080, China; lpf5356@hrbust.edu.cn; 4 Hefei National Laboratory, Hefei 230088, China

**Keywords:** adaptive Kalman filter, multi-source time fusion, TCXO, BeiDou Navigation Satellite System (BDS), eLoran, chip-level time reference

## Abstract

**Highlights:**

**What are the main findings?**

- A three-layer adaptive Kalman filter time-fusion method is developed by integrating a digitally temperature-compensated TCXO, BDS timing correction, and eLoran backup, enabling coordinated short-term prediction, medium-term correction, and long-term abnormal-state compensation.
- Experimental results demonstrate that the proposed method significantly reduces timing errors while improving timing continuity and robustness under BDS degradation and outage conditions.

**What are the implications of the main findings?**

- The proposed method enhances the accuracy, continuity, and robustness of chip-level time references in complex environments where BDS/GNSS signals may degrade, be blocked, or become unavailable.
- The three-layer fusion framework provides a practical technical route for reliable terrestrial timing and synchronization in satellite navigation receivers, communication systems, and other time-sensitive applications.

**Abstract:**

This paper proposes a three-layer adaptive Kalman filter-based multi-source time fusion method for constructing a high-precision, continuous, and robust chip-level time reference. A digitally temperature-compensated TCXO is used as the short-term local time reference, and the Allan variance is introduced to characterize oscillator frequency stability and model the process-noise covariance. For medium-term correction, BeiDou observations are fused with oscillator prediction through an adaptive Kalman filter. A reliability score based on $C/N_{0}$, DOP, pseudo-range residuals, and other quality indicators is used to dynamically adjust the observation-noise covariance and Kalman gain. When BeiDou signals become unreliable or unavailable, eLoran is introduced as a backup timing source to maintain continuous output. In addition, Kalman filter residuals are fed back to the TCXO temperature-compensation module, forming a closed-loop correction mechanism to suppress residual frequency drift and accumulated timing errors. Experimental results show that the proposed method significantly reduces timing errors and improves continuity, stability, and recovery capability under BeiDou degradation and outage conditions.

## 1. Introduction

High-precision time references are widely employed in modern technological systems and constitute one of the core technologies for satellite navigation receivers, communication base stations, aerospace positioning, and control systems [1]. These applications impose stringent requirements on the continuity, accuracy, and robustness of time outputs, where even short-term fluctuations or long-term drifts may degrade system performance or even lead to safety risks. Therefore, providing a high-precision, continuous, and reliable time reference has become a fundamental challenge for various receivers and time-sensitive systems.

However, a single time source cannot simultaneously satisfy the requirements for short-term stability, long-term accuracy, and fault tolerance in complex environments. Local oscillators, such as temperature-compensated crystal oscillators (TCXOs) and oven-controlled crystal oscillators (OCXOs), exhibit excellent short-term frequency stability but suffer from significant long-term timing drift due to temperature variations, aging, and power-supply disturbances [2]. Global Navigation Satellite System (GNSS) signals, such as those from the BeiDou Navigation Satellite System (BDS) and the Global Positioning System (GPS), can provide external timing observations to constrain the long-term drift of the local clock. Nevertheless, GNSS signals are vulnerable to signal blockage, multipath effects, and attenuation in urban canyons, indoor environments, and electromagnetically disturbed scenarios, resulting in unreliable instantaneous observations. In this study, a single-receiver BDS timing-observation model is adopted, and real observation data are used to extract signal-quality indicators and construct the corresponding timing observations. The model does not involve common-view, all-in-view, or precise point positioning time transfer and is not intended to assess absolute timing accuracy relative to BDT or UTC. Low-frequency terrestrial navigation systems, such as enhanced Loran (eLoran), provide wide-area, GNSS-independent coverage and are generally more resistant to interference than GNSS [3]. However, eLoran timing accuracy depends strongly on transmitter synchronization, propagation-delay calibration, time-of-arrival estimation, and additional secondary factor correction. Although the eLoran observation update interval adopted in this study is relatively long and its instantaneous accuracy is limited, eLoran can serve as a redundant external timing reference under GNSS-degraded or GNSS-denied conditions. Consequently, the limitations of individual timing sources necessitate multi-source time fusion techniques to exploit their complementary advantages [4].

Accordingly, multi-source time fusion has attracted increasing attention in recent years. Existing approaches mainly include weighted averaging, Kalman filtering and its variants, and GNSS/eLoran integrated timing strategies. Weighted averaging is computationally efficient but cannot effectively adapt to dynamic changes in signal quality and is susceptible to abnormal observations [5]. Standard Kalman filtering enables optimal fusion through state prediction and observation correction while dynamically allocating source weights according to uncertainty estimates; however, its performance strongly depends on accurate system models and noise statistics, making practical implementation relatively complex [6,7]. GNSS/eLoran redundancy strategies improve system robustness but generally pay insufficient attention to short-term oscillator drift prediction, temperature compensation, and closed-loop correction mechanisms [8]. Consequently, maintaining both high accuracy and continuous timing output under signal degradation or outages remains a significant challenge. Achieving an effective balance among short-term stability, long-term accuracy, and robustness under abnormal conditions has therefore become a key issue in multi-source time fusion research.

**Figure 1:**
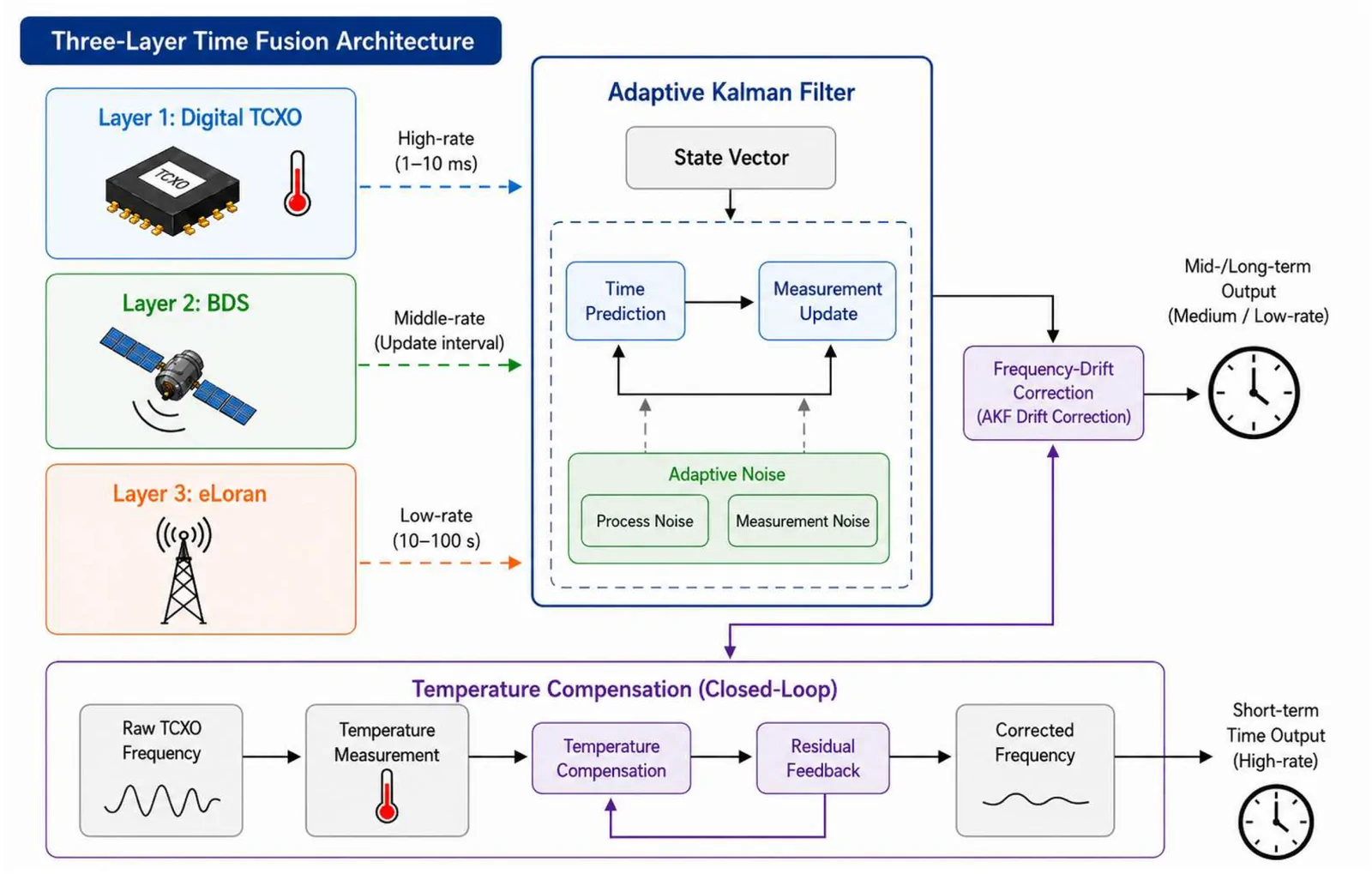
Overall architecture of the three-layer adaptive Kalman filter-based multi-source time fusion method.

To address these challenges, this paper proposes a three-layer adaptive Kalman filter-based multi-source time fusion method for constructing a high-precision, continuous, and robust chip-level time reference in complex environments. The overall architecture of the proposed method is shown in Figure 1. In Figure 1, the terms “High-rate,” “Middle-rate,” and “Low-rate” describe the relative update intervals of the TCXO, BDS, and eLoran layers in the proposed architecture. At the first layer, a digitally temperature-compensated TCXO serves as the short-term local reference. Short-term oscillator drift is modeled through linear state prediction and Allan variance analysis to generate continuous high-rate timing outputs [9]. The prediction residual is then fed back to the temperature-compensation module, forming a closed-loop correction mechanism that continuously suppresses oscillator drift accumulation and improves short-term stability. At the second layer, BDS observations are fused with oscillator predictions using an adaptive Kalman filter. Signal-quality indicators, including the carrier-to-noise density ratio ($C/N_{0}$), dilution of precision (DOP), pseudo-range residuals, and instantaneous timing drift, are employed to dynamically adjust the observation-noise covariance and Kalman gain. Consequently, the filter assigns greater confidence to BDS observations under favorable conditions while automatically reducing their influence when signals are degraded or blocked, thereby improving medium-term timing accuracy. At the third layer, when BDS observations become unavailable or unreliable, eLoran measurements are introduced as backup observations. Continuous time output is maintained through Kalman filter state prediction and residual correction, thereby ensuring system robustness under BDS-degraded or BDS-denied conditions. By integrating short-term oscillator prediction, medium-term BDS correction, and long-term eLoran compensation into a unified three-layer closed-loop framework [10], the proposed method enhances short-term stability, long-term accuracy, and timing continuity under abnormal signal conditions, providing a systematic and practically implementable solution for multi-source time fusion.

Compared with conventional single-source synchronization and simple multi-source fusion methods, the proposed three-layer adaptive Kalman filter framework comprehensively considers the characteristics of heterogeneous timing sources, short-term stochastic oscillator drift, and environmental disturbances. By combining digital temperature compensation, Allan variance-based process noise modeling, adaptive BDS observation weighting, and residual-feedback closed-loop correction, the proposed approach effectively balances short-term stability, long-term accuracy, and robustness under adverse conditions. Experimental results demonstrate that the proposed method provides continuous, accurate, and reliable chip-level time references under multi-source fusion scenarios, offering both theoretical support and practical feasibility for satellite navigation receivers, communication systems, and other time-critical applications.

## 2. Oscillator Module

### 2.1. Fundamental Model of Oscillator Output

Let the nominal frequency of the crystal oscillator at the reference temperature $T_{0}$ be $f_{0}$, and let its actual output be f(t). The relative frequency deviation is defined as

(1) $$(t)=\frac{f\left(t\right)-f_{0}}{f_{0}}$$

where y(t) includes the short-term random drift of the crystal oscillator itself, temperature effects, aging drift, power-supply disturbances, and other factors [11].

For a discrete sampling process with a sampling interval of $\tau_{0}$, the average frequency deviation over the i-th sampling interval is [12]

(2) $${\bar{y}}_{i}=\frac{1}{\tau_{0}}\int_{i\tau_{0}}^{\left(i+1\right)\tau_{0}}y\left(t\right)dt$$

The output frequency of the crystal oscillator over a short time scale, such as 0.1–1 s, is mainly affected by two types of factors [2]: one is slow linear drift caused by short-term aging or temperature variation of the crystal oscillator itself, which can be represented by a continuous linear function; the other is high-frequency random noise, such as thermal noise and circuit noise, whose mean is close to zero and appears as a random perturbation. Therefore, the crystal oscillator frequency over a sampling interval on a short time scale can be approximated as linear drift:

(3) $$y\left(t\right)=y_{0}+\dot{y_{i}}t+\eta (t)=y_{i}+\dot{y_{i}}\left(t-i\tau_{0}\right)+\eta (t)$$

where $y_{0}$ is the initial relative frequency deviation, $y_{i}$ is the relative frequency deviation at the starting time of the i-th sampling interval, $\dot{y_{i}}$ is the short-term drift rate, whose value is approximately constant, and η(t) denotes zero-mean random frequency noise.

From Equations (2) and (3), we have the following:

(4) $${\bar{y}}_{i}=\frac{1}{\tau_{0}}\int_{i\tau_{0}}^{\left(i+1\right)\tau_{0}}\left(y_{0}+\dot{y}t+\eta \left(t\right)\right)dt=y_{0}+\dot{y}\frac{2i+1}{2}\tau +\frac{1}{\tau_{0}}\int_{i\tau_{0}}^{\left(i+1\right)\tau_{0}}\eta \left(t\right)dt\approx y_{0}+\dot{y}\frac{2i+1}{2}\tau_{0}$$

If $\dot{y}$ is approximately unchanged within adjacent short-time windows, and the mean term of the zero-mean random noise is ignored, then

(5) $${\bar{y}}_{i+1}-{\bar{y}}_{i}\approx \dot{y_{i}}\tau_{0}$$

The Allan variance of the frequency deviation is [9]

(6) $$\sigma_{y}^{2}(\tau_{0})=\frac{1}{2}E\left[{\left({\bar{y}}_{i+1}-{\bar{y}}_{i}\right)}^{2}\right]$$

Therefore, from Equations (5) and (6), we have

(7) $$\sigma_{y}^{2}(\tau )\approx \frac{1}{2}{\dot{y}}^{2}\tau_{0}^{2}$$

Thus, the short-term frequency drift rate can be approximately expressed as

(8) $$|\;\dot{y}|\approx \frac{\sqrt{2\sigma_{y}^{2}(\tau_{0})}}{\tau_{0}}=\frac{\sqrt{2}\sigma_{y}(\tau_{0})}{\tau_{0}}$$

Equations (7) and (8) show that, on a short time scale, the Allan variance can be used to characterize the variation degree of adjacent samples of the crystal oscillator’s relative frequency deviation and can provide a basis for the subsequent estimation of the crystal oscillator drift rate and modeling of the process-noise covariance in Kalman filtering. Therefore, the short-term time error of the crystal oscillator can be further described using a linear state prediction model [2]:

(9) $$\delta t_{k+1}=\delta t_{k}+y_{k}\Delta t+\frac{1}{2}{\dot{y}}_{k}\Delta t^{2}+w_{\delta t,k}$$

(10) $$y_{k+1}=y_{k}+{\dot{y}}_{k}\Delta t+w_{y,k}$$

(11) $${\dot{y}}_{k+1}={\dot{y}}_{k}+w_{\dot{y},k}$$

where $\delta t_{k}$ is the phase error of the local crystal oscillator time relative to the external reference time, $y_{k}$ is the relative frequency deviation, ${\dot{y}}_{k}$ is the short-term frequency drift rate, and $w_{\delta t,k}$, $w_{y,k}$, and $w_{\dot{y},k}$ are process noises caused by random noise of the crystal oscillator, residual temperature effects, and aging disturbances.

### 2.2. Digital Temperature Compensation Model

Since the frequency of a crystal oscillator varies with temperature, when the temperature $T_{k}$ deviates from the reference temperature $T_{0}$, the temperature deviation is defined as $\Delta T_{k}=T_{k}-T_{0}$, and the frequency variation can be approximately represented by a polynomial as follows [13]:

(12) $$f_{XO}(t)=f_{0}\left(1+\alpha \Delta T_{k}+\beta {\Delta T_{k}}^{2}\right)+\eta (t)$$

(13) $$y_{T}\left(T_{k}\right)=\alpha \Delta T_{k}+\beta \Delta T_{k}^{2}$$

where $y_{T}\left(T_{k}\right)$ is the relative frequency deviation caused by the crystal oscillator temperature, $\alpha \Delta T_{k}$ is the first-order linear term, and α is the first-order temperature coefficient of the crystal oscillator. This term reflects the direct influence of small temperature changes on frequency and is the main contribution to temperature drift.

For high-precision crystal oscillators, the curve of frequency variation with temperature is not strictly linear, and curvature or asymmetry may occur. Therefore, the second-order quadratic term $\beta \Delta T_{k}^{2}$ is used to correct this nonlinear characteristic, making temperature compensation more accurate. Here, β is the second-order temperature coefficient.

From Equations (9) to (12), it can be seen that the total frequency offset of the crystal oscillator output caused by temperature variation can be used for digital temperature compensation. By measuring the temperature $T_{k}$, calculating the offset, and applying correction, the short-term stability of the crystal oscillator and the overall accuracy of the time reference can be improved [14]. The relative frequency deviation after digital temperature compensation is

(14) $$y_{comp}\left(t\right)=\frac{f_{XO}\left(t\right)-f_{0}}{f_{0}}-\hat{\alpha }\Delta T_{k}-\hat{\beta }\Delta T_{k}^{2}$$

where $\hat{\alpha }$ and $\hat{\beta }$ are the estimated temperature-compensation coefficients. The corresponding compensated frequency is

(15) $$f_{comp}\left(t\right)=f_{0}\left(1+y_{comp}\left(t\right)\right)$$

This output removes the linear and quadratic drift components caused by temperature from the original frequency output, so that the crystal oscillator output no longer produces systematic offsets due to temperature variation, thereby maintaining stability. These effects may require piecewise, higher-order, or hysteresis-aware models under wider or rapidly varying temperature conditions [14].

At the same time, since high-frequency random jitter η(t) still exists in the crystal oscillator output, this part of the short-term random noise cannot be eliminated through temperature compensation. Therefore, in the subsequent filtering process, the short-term random noise η(t) of the crystal oscillator is regarded as the main source of process noise. Retaining η(t) provides realistic process uncertainty, enabling the filter to correctly estimate the short-term drift of the crystal oscillator and ensuring short-term stability and robustness.

## 3. BeiDou Signal and eLoran Signal Modules

### 3.1. BeiDou Signal Modeling and Reliability Evaluation

The BeiDou Navigation Satellite System (BDS) can provide high-precision timing information; however, satellite-to-ground timing links remain susceptible to ionospheric and tropospheric delays, signal blockage, and multipath interference, thereby reducing observation accuracy [15]. Meanwhile, multipath- and NLOS-related errors may contain non-zero biases and non-Gaussian components; consequently, actual GNSS observation errors affected by multipath and NLOS propagation do not necessarily follow a zero-mean Gaussian distribution [16]. Therefore, the time observation received from BeiDou can be expressed as follows:

(16) $$z_{BDS\;,\;k}=\delta t_{k}+b_{BDS,\;k}+\varepsilon_{BDS\;,\;k}$$

where $z_{BDS\;,\;k}$ is the local time error observation provided by BeiDou, $\delta t_{k}$ is the local time phase error, $b_{BDS,\;k}$ is the equivalent residual bias of the BeiDou observation link, and $\varepsilon_{BDS,k}$ is the remaining short-term random residual after separating the slowly varying bias. Specifically, $b_{BDS,k}$ accounts for incompletely corrected ionospheric and tropospheric delays, receiver- and antenna-cable-related hardware delays, and persistent multipath or NLOS components.

(17) $$\varepsilon_{BDS\;,\;k}|\rho_{BDS,k}\approx N(0,R_{BDS,k})$$

where $\rho_{BDS,k}$ is the composite BDS reliability score defined below. Equation (17) represents only a conditional working approximation for the remaining short-term random residual after separating the equivalent slowly varying link bias; it does not imply that the complete BDS timing-link error always follows a zero-mean Gaussian distribution under multipath, signal blockage, interference, or NLOS propagation conditions. The observation-noise covariance $R_{BDS,k}$ is dynamically adjusted according to $\rho_{BDS,k}$.

To dynamically adjust the weight of the BDS observation in the Kalman filter (KF), a comprehensive reliability score $\rho_{BDS,k}$ is introduced as [17]

(18) $$\rho_{BDS,k}=w_{1}{\tilde{C}}_{N0,k}+w_{2}{\tilde{D}}_{k}+w_{3}{\tilde{r}}_{PR,k}+w_{4}{\tilde{d}}_{inst,k}$$

subject to

(19) $$0\leq \rho_{BDS,k}\leq 1\;\;\;,\;\;\;\sum_{i=1}^{4}w_{i}=1$$

where the carrier-to-noise density ratio indicator ${\tilde{C}}_{N0,k}$ reflects the ratio between the signal strength and the receiver noise and therefore directly affects the measurement accuracy [18]. The dilution-of-precision indicator ${\tilde{D}}_{k}$ describes the effect of the geometric distribution of the satellites relative to the receiver on the time solution. A more favorable satellite geometry generally results in a smaller solution error. The pseudo-range residual indicator ${\tilde{r}}_{PR,k}$ represents the deviation between the observed value and the theoretically calculated value and can therefore directly quantify measurement consistency. The instantaneous drift indicator ${\tilde{d}}_{inst,k}$ reflects the short-term stability of the receiver time output. The coefficients $w_{i}$ are weighting factors used to combine these physical indicators into an overall reliability score, thereby dynamically adjusting the BDS observation weight in the KF.

Since the carrier-to-noise density ratio is a positive indicator, whereas DOP, the pseudo-range residual, and the instantaneous drift are negative indicators, the indicators are normalized in a consistent direction as follows. This ensures that $\rho_{BDS,k}\in \left[0,1\right]$, with a larger value indicating a more reliable observation:

(20) $${\tilde{C}}_{N0,k}=\frac{C/N_{0,k}-C/N_{0,min}}{C/N_{0,max}-C/N_{0,min}}$$

(21) $$\{\begin{array}{c} {\tilde{D}}_{k}=1-\frac{DOP_{k}-DOP_{min}}{DOP_{max}-DOP_{min}}\\ {\tilde{r}}_{PR,k}=1-\frac{r_{PR,k}-r_{PR,min}}{r_{PR,max}-r_{PR,min}}\\ {\tilde{d}}_{inst,k}=1-\frac{d_{inst,k}-d_{inst,min}}{d_{inst,max}-d_{inst,min}} \end{array}$$

Accordingly, the BDS observation-noise covariance can be dynamically adjusted according to the reliability score:

(22) $$R_{BDS,k}=\frac{R_{BDS,0}}{\rho_{BDS,k}+\epsilon_{0}}$$

where $R_{BDS,k}$ is the nominal BDS observation-noise covariance, and $\epsilon_{0}$ is a small positive constant introduced to prevent the denominator from becoming zero. This adaptive covariance adjustment reduces the influence of unreliable observations but does not establish the Gaussianity of the underlying BDS errors or fully represent abrupt biased and heavy-tailed errors.

### 3.2. eLoran Signal Characteristics and Modeling

The eLoran signal is a time-synchronization signal provided by a low-frequency, long-wave radio navigation system and is characterized by wide-area coverage and strong resistance to radio-frequency interference. However, its propagation delay and additional secondary factor (ASF) may be affected by the propagation path, ground conductivity, terrain, weather, and seasonal variations, and may therefore contain non-zero biases and temporally correlated components [19,20]. In addition, atmospheric noise in the Loran frequency band may contain large-amplitude, short-duration impulses, which can distort the received waveform and cause abnormal time-of-arrival (TOA) estimates or cycle-selection errors [21]. Therefore, the complete error in actual eLoran observations cannot simply be regarded as independent, zero-mean Gaussian white noise with a fixed variance. In a multi-source time-fusion system, eLoran can serve as a backup observation source when the BDS signal is unavailable or unreliable, thereby providing the system with an independent external time reference. The eLoran observation can be expressed as [3]

(23) $$z_{LW,k}=\delta t_{k}+b_{LW,k}+\varepsilon_{LW,k}$$

where $z_{LW,k}$ denotes the observation of the local clock error provided by eLoran, $b_{LW,k}$ is the equivalent residual propagation-delay bias of the eLoran link, and $\varepsilon_{LW,k}$ is the remaining short-term measurement residual after separating the slowly varying bias [22]. Specifically, $b_{LW,k}$ accounts for the residual transmitting-station synchronization bias, propagation-delay residual, ASF residual, and receiver-related hardware bias after nominal calibration.

Under the nominal conditions considered in the controlled simulation, the remaining short-term residual is described using the following working approximation:

(24) $$\varepsilon_{LW,k}\approx N(0,R_{LW})$$

The eLoran observation noise incorporates several physical error sources, including the small time-synchronization error of the eLoran transmitting station, which is generally on the order of nanoseconds to microseconds; the limited measurement accuracy of the receiver in determining the pulse time of arrival (TOA); and propagation-related errors such as ground-wave propagation delay, atmospheric delay, and multipath effects [23]. Therefore, the eLoran observation noise can be further decomposed as

(25) $$\{\begin{array}{c} \;\;b_{LW,k}=b_{sync,k}^{\left(s\right)}+b_{prop,k}+b_{ASF,k}+b_{hw,k}\\ \;\varepsilon_{LW,k}=\varepsilon_{sync,k}^{\left(s\right)}+\varepsilon_{rec,k}+\varepsilon_{TOA,k}\;\;\;\;\;\;\;\;\;\;\;\;\;\;\;\;\; \end{array}$$

where $b_{sync,k}^{\left(s\right)}$ denotes the slowly varying residual synchronization bias of the transmitting station, $b_{prop,k}$ denotes the residual ground-wave propagation delay, $b_{ASF,k}$ denotes the residual error after ASF correction, and $b_{hw,k}$ denotes the receiver-related hardware-delay bias. These slowly varying components are aggregated into the equivalent bias state $b_{LW,k}$.

The terms $\varepsilon_{sync,k}^{\left(s\right)}$, $\varepsilon_{TOA,k}\;$, and $\varepsilon_{rec,k}$ denote the short-term transmitting-time jitter, TOA-estimation noise, and receiver measurement noise, respectively. These fast residual components constitute $\varepsilon_{LW,k}$.

Since the above error components originate from the transmitter, the signal-propagation process, and the receiver, respectively, they have different generation mechanisms and are weakly correlated over short time scales. Therefore, they can be approximately treated as mutually independent random variables. Therefore, the total observation-noise covariance can be obtained through additive variance composition [24]:

(26) $$R_{LW}=\sigma_{sync,j}^{2}+\sigma_{TOA}^{2}+\sigma_{rec}^{2}$$

The propagation-delay and ASF uncertainties are not included in $R_{LW}$, because they are represented by the bias state $b_{LW,k}$ and its corresponding process-noise covariance.

The variance of each error component can be obtained through experimental measurements or statistical analysis of historical data. Since eLoran is a low-frequency, long-wave system with a low update rate, its short-term noise characteristics are considered approximately stable.

Therefore, in the finite-duration KF framework developed in this study, the eLoran observation noise can be modeled directly using a fixed covariance to simplify the KF measurement-update calculation, without frequently calculating a time-varying $R_{LW,k}$:

(27) $$R_{LW,k}=R_{LW}$$

## 4. Kalman Filter

### 4.1. KF State-Space Model

To estimate the internal master time reference of the chip and the short-term frequency states of the local oscillator, existing clock-disciplining and time-scale algorithms commonly use the time-phase error, relative frequency offset, and frequency-drift rate as the fundamental state variables, and employ a local quadratic polynomial to describe the temporal evolution of the clock-phase error [24]. On this basis, the equivalent slowly varying residual biases of the BDS and eLoran timing links are further incorporated into the state vector:

(28) $$x_{k}={\left[\begin{array}{ccc} \delta t_{k} & y_{k} & \begin{array}{ccc} {\dot{y}}_{k} & b_{BDS,k} & b_{LW,k} \end{array} \end{array}\right]}^{T}$$

where $\;\delta t_{k}$ denotes the phase error of the chip’s local time relative to the external reference time; $y_{k}$ denotes the residual fractional-frequency offset of the TCXO after digital temperature compensation; and ${\dot{y}}_{k}$ denotes its frequency-drift rate. The state $b_{BDS,k}$ denotes the equivalent slowly varying residual bias of the BDS timing link, including incompletely corrected atmospheric-delay residuals [16], receiver- and antenna-cable-related hardware delays, and persistent multipath or NLOS components. The state $b_{LW,k}$ denotes the equivalent slowly varying residual bias of the eLoran timing link, including residual transmitting-station synchronization bias, propagation-delay residual, ASF residual, and receiver-related hardware-delay bias [14].

According to Equation (28), the states $\delta t_{k}$, $y_{k}$, and ${\dot{y}}_{k}$ constitute the phase-error, fractional-frequency-offset, and frequency-drift-rate model of the oscillator-based local clock, respectively. The local time-phase error is propagated through the integration of the fractional-frequency offset, while the fractional-frequency offset is updated through the integration of the frequency-drift rate. Over a short time window, the frequency-drift rate is approximated as a slowly varying state. The states $b_{BDS,k}$ and $b_{LW,k}$ are modeled as first-order Gauss–Markov processes with finite correlation times to characterize the temporal correlations and slowly varying behavior of the corresponding link biases [25]. 

In this model, $y_{k}$ is explicitly defined as the residual fractional-frequency offset after digital temperature compensation and therefore already contains the remaining frequency error after compensation. Consequently, the temperature-compensation residual is not introduced as an additional Kalman-filter state. The temperature-compensation and residual-feedback modules remain outside the Kalman-filter state vector. Their correction is reflected in the subsequent compensated-frequency sequence and hence in the evolution of $y_{k}$. Accordingly, the augmented state vector is defined in Equation (28).

Over a short time scale, the oscillator frequency offset can be approximated using a low-order linear stochastic model. Let the Kalman filter prediction interval be Δt. The state prediction equation is

(29) $$x_{k+1}^{-}=F_{k}x_{k}^{+}+\omega_{k}\;\;\;,\;\;\;\omega_{k}∼N\left(0\;,Q_{k}\right)$$

where $x_{k+1}^{-}$ is the predicted state, $x_{k}^{+}$ is the updated state at the previous time step, and $\omega_{k}$ is the process noise representing the short-term random drift of the oscillator. In the three-state model, $\omega_{k}$ can be expressed as

(30) $$\omega_{k}=\left[\begin{array}{c} \begin{array}{c} \omega_{\delta t,k}\\ \omega_{y,k}\\ \omega_{\dot{y},k} \end{array}\\ \omega_{BDS,k}\\ \omega_{LW,k} \end{array}\right]$$

where $\omega_{\delta t,k}$ denotes the process disturbance of the local time-phase error during the prediction interval, mainly arising from the integration of oscillator-frequency noise and unmodeled timing disturbances; $\omega_{y,k}$ denotes the random variation in the residual fractional-frequency-offset prediction; and $\omega_{\dot{y},k}$ represents the uncertainty in the frequency-drift-rate state. Furthermore, $\omega_{BDS,k}$ and $\omega_{LW,k}$ represent random variations in the equivalent slowly varying residual biases of the BDS and eLoran timing links, respectively.

Considering the integrated effect of the residual temperature-compensation frequency offset $b_{T,k}$ on the time error, the state-transition matrix can be written as

(31) $$F_{k}=\left[\begin{array}{c} \begin{array}{ccc} 1 & \Delta t & \begin{array}{ccc} \frac{1}{2}\Delta t^{2} & 0 & 0 \end{array} \end{array}\\ \begin{array}{ccc} 0 & 1 & \begin{array}{ccc} \Delta t & 0 & 0 \end{array} \end{array}\\ \begin{array}{ccc} 0 & 0 & \begin{array}{ccc} 1 & 0 & 0 \end{array}\\ 0 & 0 & \begin{array}{ccc} 0 & \phi_{BDS} & 0 \end{array}\\ 0 & 0 & \begin{array}{ccc} 0 & 0 & \phi_{LW} \end{array} \end{array} \end{array}\right]$$

In Equation (28), $y_{k}$ is dimensionless, ${\dot{y}}_{k}$ has units of $s^{-1}$, and $\delta t_{k}$, $b_{BDS,k}$ and $b_{LW,k}$ have units of time. Therefore, Δt has units of time and is dimensionally consistent with $\;\delta t_{k}$. The BDS and eLoran link biases are not integrated into the local clock-phase state; instead, they enter the corresponding measurement equations additively. This distinction prevents source-specific link biases from being interpreted as components of the local oscillator frequency error.

The coefficients $\phi_{BDS}$ and $\phi_{LW}$ denote the state-retention coefficients of the BDS and eLoran link biases, respectively. In the revised model, these slowly varying bias states are described as finite-correlation first-order Gauss–Markov processes [25]. Their state-retention coefficients are defined below, where $\phi_{BDS}$, and $\phi_{LW}$ denote the state-retention coefficients of the BeiDou link bias and eLoran propagation, respectively. When $\phi_{s}=1$ and the corresponding process-noise variance is greater than zero, the state can be approximately modelled as a random-walk process. When $0<\phi_{s}<1$, the state can be modeled as a first-order Gauss–Markov process [25] to characterize the finite temporal correlation and mean-reverting behavior of the bias. For a first-order Gauss–Markov model, the state-retention coefficient is commonly expressed as

(32) $$\phi_{s}=\exp\left(-\frac{\Delta t}{\tau_{s}}\right)\;\;\;s\in \left\{BDS,LW\right\}$$

where $\tau_{s}$ is the correlation time constant of the corresponding bias state.

For high-quality oscillators, such as TCXOs and OCXOs, short-term drift is mainly caused by white frequency noise and random-walk frequency noise. These noise components vary slowly over time scales of several seconds or less, allowing the drift trend to be approximated as linear. However, when the prediction interval exceeds several seconds or even tens of seconds, oscillator temperature variation, aging, and environmental disturbances can significantly affect the frequency output, causing the linear assumption to become invalid [2]. Conversely, if the prediction interval is too short, prediction updates become excessively frequent, increasing the computational burden while providing only limited performance improvement. Therefore, Δt should be selected to balance prediction accuracy and computational efficiency while maintaining the validity of the short-term linear approximation.

### 4.2. Process-Noise Covariance Matrix

In the Kalman filter, the process-noise covariance matrix $Q_{k}$ represents the uncertainty of the system states during the prediction interval. The short-term frequency drift and random jitter of the oscillator are the main sources of this uncertainty [12]. In this study, the Allan variance $\sigma_{y}^{2}(\tau )$ is used to quantify the magnitude of oscillator frequency fluctuations over the sampling interval τ [26], and the process-noise covariance matrix is dynamically tuned accordingly.

According to Equations (28)–(31), for the augmented state vector, the process-noise covariance matrix can be written as

(33) $$Q_{k}=\left[\begin{array}{cc} Q_{osc,k} & 0\\ 0 & Q_{bias,k} \end{array}\right]$$

where $Q_{osc,k}$ denotes the process-noise covariance associated with the local clock-phase error, residual fractional-frequency offset, and frequency-drift rate, whereas $Q_{bias,k}$ denotes the process-noise covariance associated with the slowly varying residual biases of the BDS and eLoran timing links.

The oscillator-related covariance matrix can be expressed as [27]

(34) $$Q_{osc,k}=\left[\begin{array}{ccc} q_{t} & q_{tf} & q_{td}\\ q_{ft} & q_{f} & q_{fd}\\ q_{dt} & q_{df} & q_{d} \end{array}\right]$$

where $q_{f}$ represents the uncertainty of the relative frequency offset of the oscillator, $q_{t}$ represents the accumulated uncertainty caused by integrating the frequency offset into the time phase over the prediction interval Δt, and $q_{d}$ represents the uncertainty of the frequency drift rate. The cross terms $q_{tf}$ and others represent covariances, namely,

(35) $$\{\begin{array}{c} q_{t}=Var\left(\omega_{t}\right)\\ q_{f}=Var\left(\omega_{f}\right)\\ q_{d}=Var\left(\omega_{d}\right) \end{array}$$

and

(36) $$\{\begin{array}{c} q_{tf}=q_{ft}=Cov\left(\omega_{t}\;,\;\omega_{f}\right)\\ q_{td}=q_{dt}=Cov\left(\omega_{t}\;,\;\omega_{d}\right)\\ q_{df}=q_{fd}=Cov\left(\omega_{d}\;,\;\omega_{f}\right) \end{array}$$

where Var(x) denotes the variance of variable x, and Cov(a , b) denotes the covariance between variables a and b.

Because the same oscillator noise source can simultaneously affect the time phase, frequency offset, and frequency drift rate, these states may be statistically correlated. For example, frequency-offset noise is integrated into a time error over one prediction interval Δt. Therefore, $\omega_{t}$ is not completely independent, and $q_{tf}$ should not simply be set to zero.

In the three-state oscillator model, the Allan variance is first used to estimate the uncertainty of the oscillator frequency drift rate. According to the definition of Allan variance [9]

(37) $$\sigma_{y}^{2}\left(\tau \right)=\frac{1}{2}E\left[{\left({\bar{y}}_{i+1}-{\bar{y}}_{i}\right)}^{2}\right]$$

where ${\bar{y}}_{i}$ and ${\bar{y}}_{i+1}$ denote the mean frequency offsets over two adjacent averaging intervals of duration τ. If the rate of change between two adjacent mean frequency offsets is approximated as the short-term oscillator frequency drift rate, then according to Equation (8),

(38) $${\dot{y}}_{k}\approx \frac{{\bar{y}}_{i+1}-{\bar{y}}_{i}}{\tau }$$

Therefore, the process-noise variance of the frequency-drift-rate state can be approximated as

(39) $$q_{d}=Var(\omega_{\dot{y},k})\approx \frac{2\sigma_{y}^{2}\left(\tau_{0}\right)}{{\tau_{0}}^{2}}$$

During the prediction interval Δt, the frequency-drift-rate noise is integrated into frequency-offset noise:

(40) $$\omega_{y,k}\approx \omega_{\dot{y},k}\Delta t$$

Therefore,

(41) $$q_{f}=Var\left(\omega_{y,k}\right)\approx \Delta t^{2}q_{d}=2\sigma_{y}^{2}\left(\tau_{0}\right){\left(\frac{\Delta t}{\tau_{0}}\right)}^{2}$$

Meanwhile, the frequency-drift-rate noise is further integrated into time-phase-error noise:

(42) $$\omega_{t}\approx \frac{1}{2}{\omega_{\dot{y},k}}_{k}\Delta t^{2}$$

Therefore,

(43) $$q_{t}=Var\left(\omega_{\delta t,k}\right)\approx \frac{1}{4}\Delta t^{4}q_{d}=\frac{1}{2}\sigma_{y}^{2}\left(\tau_{0}\right){\left(\frac{\Delta t^{2}}{\tau_{0}}\right)}^{2}$$

Accordingly, the process-noise covariance matrix of the oscillator states is

(44) $$Q_{osc,k}=q_{d}\left[\begin{array}{ccc} \frac{1}{4}\Delta t^{4} & \frac{1}{2}\Delta t^{3} & \frac{1}{2}\Delta t^{2}\\ \frac{1}{2}\Delta t^{3} & \Delta t^{2} & \Delta t\\ \frac{1}{2}\Delta t^{2} & \Delta t & 1 \end{array}\right]\approx \frac{2\sigma_{y}^{2}\left(\tau_{0}\right)}{{\tau_{0}}^{2}}\left[\begin{array}{ccc} \frac{1}{4}\Delta t^{4} & \frac{1}{2}\Delta t^{3} & \frac{1}{2}\Delta t^{2}\\ \frac{1}{2}\Delta t^{3} & \Delta t^{2} & \Delta t\\ \frac{1}{2}\Delta t^{2} & \Delta t & 1 \end{array}\right]$$

In practical implementations, the Allan-deviation curve of the compensated TCXO can be used to identify the dominant oscillator-noise types over different averaging intervals. If necessary, different weighting coefficients may be introduced for white frequency noise, flicker frequency noise, and random-walk frequency noise. In the present model, however, Equation (44) is retained as a short-term engineering approximation for constructing the oscillator process-noise covariance from the Allan variance.

The BDS and eLoran link-bias states arise from different signal-generation mechanisms, propagation paths, and receiving processes. In the absence of evidence of common driving factors, their process disturbances are approximated as mutually independent. The process-noise covariance matrix of the two bias states is therefore written as [10,14]

(45) $$Q_{bias,k}=\left[\begin{array}{cc} q_{BDS} & 0\\ 0 & q_{LW} \end{array}\right]$$

where $q_{BDS}$ denotes the process-noise variance used to describe random variations in the equivalent slowly varying residual bias $b_{BDS,k}$ of the BDS timing link, and $q_{LW}$ denotes the process-noise variance used to describe random variations in the equivalent slowly varying residual bias $b_{LW,k}$ of the eLoran timing link.

It should be emphasized that $Q_{bias,k}$ characterizes only the temporal-evolution uncertainty of the slowly varying link-bias states. The remaining short-term BDS and eLoran measurement residuals are represented separately by $R_{BDS,k}$ and $R_{LW,k}$ in the measurement equations. In particular, persistent multipath or NLOS components are included in $b_{BDS,k}$, whereas propagation-delay and ASF residuals are included in $b_{LW,k}$. These components are therefore not included again in the corresponding measurement-noise covariance, thereby avoiding duplicate modeling between the process-noise and measurement-noise terms.

### 4.3. Measurement Equation

Let the time observation provided by BDS or eLoran be $z_{k}$. The measurement equation can then be written as

(46) $$z_{k}=H_{k}x_{k}+\varepsilon_{k}$$

where $H_{k}$ is the measurement matrix corresponding to the selected observation source, and $\varepsilon_{k}$ denotes the remaining short-term measurement residual after the source-specific slowly varying link bias has been separated. The statistical model of $\varepsilon_{k}$ depends on the currently selected observation source.

When the observation source is BDS, the measurement equation is [7]

(47) $$z_{BDS,k}=H_{BDS}x_{k}+\varepsilon_{BDS,k}$$

(48) $$H_{BDS}=\left[1\;\;\;0\;\;\;0\;\;\;1\;\;\;0\right]$$

This equation indicates that the BDS observation mainly corresponds to the local time phase error $\Delta t_{k}$ of the chip and also contains the equivalent BDS link bias $b_{BDS,k}$. The BDS measurement noise satisfies

(49) $$\varepsilon_{BDS,k}|\rho_{BDS,k}∼N(0\;,\;R_{BDS,k})$$

where $\rho_{BDS,k}$ is the composite BDS reliability score defined in Equation (18), and $R_{BDS,k}$ is dynamically adjusted according to Equation (22). When the BDS signal quality is high, $\;\rho_{BDS,k}$ increases and $R_{BDS,k}$ decreases, so that a larger weight is assigned to the BDS observation. When the BDS signal is degraded by blockage, multipath, interference, or NLOS propagation, $\rho_{BDS,k}$ decreases and $R_{BDS,k}$ increases, thereby reducing the influence of the corresponding observation on the state estimate.

Moreover, dynamically adjusting $R_{BDS,k}$ according to the reliability score reduces the influence of degraded observations but does not constitute a statistical verification of Gaussianity.

When the BDS signal is unavailable or its reliability is below a predefined threshold, eLoran is introduced as a backup time observation source. The corresponding measurement equation is

(50) $$z_{LW,k}=H_{LW}x_{k}+\varepsilon_{LW,k}$$

(51) $$H_{LW}=\left[1\;\;\;0\;\;\;0\;\;\;0\;\;\;1\right]$$

This equation indicates that the eLoran observation mainly corresponds to the local time phase error $\Delta t_{k}$ of the chip and also contains the eLoran propagation-delay residual $b_{LW,k}$ [20]. The eLoran measurement noise satisfies

(52) $$\varepsilon_{LW,k}∼N(0\;,\;R_{LW,k})$$

where $R_{LW,k}$ is the fixed covariance defined in Equations (26) and (27), and it characterizes only short-term transmitting-time jitter, TOA-estimation noise, and receiver measurement noise. The residual propagation delay, ASF error, residual transmitting-station synchronization bias, and receiver-related hardware-delay bias are represented by $b_{LW,k}$ and its process-noise variance. Therefore, these slowly varying error components are not included again in $R_{LW,k}$.

Equation (52) is also a working approximation used under the nominal conditions of the controlled simulation, rather than a general statistical description of actual eLoran observations. Actual eLoran residuals may contain non-zero biases, temporal correlations, impulsive disturbances, outliers, or heavy-tailed components. Such effects are not fully represented by a fixed zero-mean Gaussian covariance.

If both BDS and eLoran are available at the same time, a joint measurement equation can also be constructed as

(53) $$z_{k}=\left[\begin{array}{c} z_{BDS,k}\\ z_{LW,k} \end{array}\right]=\left[\begin{array}{ccc} 1 & 0 & \begin{array}{ccc} 0 & 1 & 0 \end{array}\\ 1 & 0 & \begin{array}{ccc} 0 & 0 & 1 \end{array} \end{array}\right]x_{k}+\left[\begin{array}{c} \varepsilon_{BDS,k}\\ \varepsilon_{LW,k} \end{array}\right]$$

The joint short-term residual is approximated under the following working assumptions:

(54) $$\varepsilon_{k}∼N(0\;,\;R_{k})$$

(55) $$R_{k}=\left[\begin{array}{cc} R_{BDS,k} & 0\\ 0 & R_{LW,k} \end{array}\right]$$

Through the state-space model described above, short-term oscillator prediction, medium-term BDS correction, eLoran backup under abnormal conditions, and temperature-compensation residuals are incorporated into a unified Kalman filtering framework [8,10]. This model can not only dynamically estimate the oscillator process-noise covariance matrix $Q_{k}$ using the Allan variance, but also dynamically adjust the measurement-noise covariance matrix $R_{k}$ according to signal quality. It therefore provides a unified mathematical basis for subsequent adaptive measurement-noise adjustment, source-switching strategies, and the KF residual-feedback temperature-compensation mechanism.

### 4.4. Observability and Identifiability Analysis

Because both the BDS and eLoran measurement equations contain the sum of the local clock-phase error and a source-specific link bias, it is necessary to examine whether these states can be estimated separately. In particular, when only one external observation source is active, a single-epoch measurement cannot directly distinguish the clock-phase error from the corresponding link bias. Their separation therefore depends on their different temporal-evolution models and observations accumulated over multiple epochs.

Suppose that only one external observation source s is active, where s∈{BDS,LW}. The active substate is defined as

(56) $$\xi_{s.k}={\left[\begin{array}{ccc} \delta t_{k} & y_{k} & \begin{array}{cc} {\dot{y}}_{k} & b_{s,k} \end{array} \end{array}\right]}^{T}$$

According to Equations (31), (48), and (51), the corresponding state-transition and measurement matrices are

(57) $$F_{s}=\left[\begin{array}{c} \begin{array}{ccc} 1 & \Delta t & \begin{array}{cc} \frac{1}{2}\Delta t^{2} & 0 \end{array} \end{array}\\ \begin{array}{ccc} 0 & 1 & \begin{array}{cc} \Delta t & 0 \end{array}\\ 0 & 0 & \begin{array}{cc} 1 & 0 \end{array}\\ 0 & 0 & \begin{array}{cc} 0 & \phi_{s} \end{array} \end{array} \end{array}\right]$$

(58) $$H_{s}=\left[\begin{array}{ccc} 1 & 0 & \begin{array}{cc} 0 & 1 \end{array} \end{array}\right]$$

where $b_{s,k}$ and $\phi_{s}$ denote the slowly varying link-bias state and its state-retention coefficient for the currently active observation source, respectively.

For this four-state submodel, the observability matrix constructed from four consecutive measurement epochs is

(59) $$O_{s}=\left[\begin{array}{c} H_{s}\\ H_{s}F_{s}\\ H_{s}F_{s}^{2}\\ H_{s}F_{s}^{3} \end{array}\right]$$

Its determinant is

(60) $$\det\left(O_{s}\right)=\Delta t^{3}{\left(\phi_{s}-1\right)}^{3}$$

Therefore, when Δt>0 and $0<\phi_{s}<1,$ the observability matrix has full rank, and the active-source submodel is structurally observable over multiple measurement epochs. Under this condition, the clock-phase error, oscillator frequency states, and active link-bias state can be separated according to their different temporal-evolution characteristics.

If $\phi_{s}$ =1, the determinant becomes zero and the observability matrix is rank deficient. In this limiting case, $b_{s,k}$ becomes a constant or random-walk state, whose temporal behavior cannot be structurally distinguished from the slowly varying component of the local clock-phase error using only the summed measurement

(61) $$z_{s,k}=\delta t_{k}+b_{s,k}+\varepsilon_{s,k}$$

Accordingly, the active BDS and eLoran link biases are modeled in this study as finite-correlation first-order Gauss–Markov processes rather than unconstrained random walks. Their state-retention coefficients are determined from the correlation-time constants defined in Equation (32).

When one observation source is unavailable, the bias state associated with that source does not appear in the active measurement equation. Consequently, the inactive-source bias is not independently observable during the corresponding outage interval. It is propagated only through its first-order Gauss–Markov model and is not directly corrected by the measurement update. Once the corresponding observation source becomes available again, its bias state is corrected using the associated measurement equation.

When valid BDS and eLoran observations are simultaneously available, subtracting the two measurement equations gives

(62) $$z_{BDS,k}-z_{LW,k}=\left(b_{BDS,k}-b_{LW,k}\right)+\left(\varepsilon_{BDS,k}-\varepsilon_{LW,k}\right)$$

The common clock-phase error is eliminated from this difference, so the simultaneous observations provide a direct constraint on the relative bias between the BDS and eLoran timing links. Nevertheless, separating the absolute clock-phase error and the individual link biases still depends on their temporal models, initial state information, and observations accumulated over multiple epochs.

It should be noted that structural observability does not necessarily guarantee accurate numerical separation under all parameter settings. When $\phi_{s}$ is close to unity, the determinant of the observability matrix approaches zero, and the clock-phase and link-bias states become weakly distinguishable in practice. Therefore, the correlation coefficients $\phi_{BDS}$ and $\phi_{LW}$, the process-noise variances $q_{BDS}$ and $q_{LW}$, and the initial bias-state covariance values must be specified consistently with the controlled simulation settings. These stochastic constraints and initial information improve practical identifiability but do not replace independent physical observations.

### 4.5. Adaptive KF Measurement-Noise Adjustment

In a multi-source time-fusion system, the measurement noise of the BDS signal varies dynamically over time and therefore needs to be adaptively adjusted in the Kalman filter [7]. According to Equations (18)–(22), the BDS measurement-noise covariance $R_{BDS\;,\;k}$ is not a fixed value but is dynamically calculated according to real-time signal-quality indicators. When the BDS signal quality is high, $\rho_{BDS,k}$ approaches 1 and $R_{BDS\;,\;k}$ is relatively small. When the signal quality deteriorates, $\rho_{BDS,k}$ approaches 0 and $R_{BDS\;,\;k}$ becomes relatively large.

The reliability score reflects signal quality before the measurement update, whereas the Kalman-filter innovation reflects the consistency between the current observation and the model prediction. Therefore, the observation-source selection should consider both quantities. For an available BDS observation, the candidate BDS innovation is defined as

(63) $$v_{BDS,k}=z_{BDS,k}-H_{BDS}{\hat{x}}_{k|k-1}$$

where ${\hat{x}}_{k|k-1}$ is the predicted state at epoch k, and $H_{BDS}$ is the BDS measurement matrix defined in Equation (48).

The variance of the candidate BDS innovation is

(64) $$S_{BDS,k}=H_{BDS}P_{k|k-1}H_{BDS}+R_{BDS,k}$$

where $P_{k|k-1}$ is the predicted state-error covariance matrix. Because the magnitude of the raw innovation depends on the units and uncertainty level of the time observation, the absolute innovation is normalized by its predicted standard deviation:

(65) $$\eta_{BDS,k}=\frac{\left|v_{BDS,k}\right|}{\sqrt{S_{BDS,k}}}$$

where $\eta_{BDS,k}$ is the dimensionless normalized absolute innovation. A smaller value indicates that the BDS observation is consistent with the predicted state and its assigned uncertainty, whereas a larger value indicates possible observation degradation, an outlier, or model mismatch.

To obtain a stable baseline for BDS reliability, a sliding window containing the most recent W BDS samples accepted under nominal operating conditions is used. Let $W_{k}$ denote the index set of these samples. The mean and standard deviation of the reliability score within the window are calculated as

(66) $$\mu_{\rho ,k}=mean\{\rho_{BDS,j}:j\in W_{k}\}$$

(67) $$\sigma_{\rho ,k}=std\{\rho_{BDS,j}:j\in W_{k}\}$$

The baseline reliability threshold is then defined as

(68) $$\rho_{th,base,k}=clip\left(\mu_{\rho ,k}-\kappa \cdot \sigma_{\rho ,k},0,1\right)\;\;,\;\;\;\kappa >0$$

where κ is a dimensionless parameter controlling the conservativeness of the reliability criterion, and clip(a,0, 1)=min[1,max(a,0)]. 

A larger value of κ produces a lower baseline threshold and therefore a more permissive BDS acceptance criterion. Conversely, a smaller value of κ produces a stricter criterion.

The normalized-innovation reference is determined using the same window of BDS samples accepted under nominal conditions. Its mean and standard deviation are calculated as

(69) $$\mu_{\eta ,k}=mean\{\eta_{BDS,j}:j\in W_{k}\}$$

(70) $$\sigma_{\eta ,k}=std\{\eta_{BDS,j}:j\in W_{k}\}$$

The target normalized-innovation level is then defined as

(71) $$\eta_{target,k}=\mu_{\eta ,k}+\alpha \sigma_{\eta ,k}$$

where α is a dimensionless safety factor used to tolerate normal short-term innovation fluctuations.

The reliability threshold is further adjusted according to the difference between the current normalized innovation and its target level [28]:

(72) $$\rho_{th,k}^{*}=\rho_{th\;,\;base}+\gamma \cdot (\eta_{BDS,k}-\eta_{target,k})$$

where γ is a dimensionless adjustment coefficient. Because both $\eta_{BDS,k}$ and $\eta_{target,k}$ are dimensionless, Equation (72) is dimensionally consistent. When the current BDS innovation is larger than the expected level, the reliability threshold increases and the BDS acceptance criterion becomes stricter. When the innovation is consistent with or lower than its expected level, the threshold remains close to or below its baseline value.

The adjusted threshold is constrained to the interval [0,1], as follows:

(73) $$\rho_{th,k}=\mathrm{clip}\left(\rho_{th,k}^{*},0,1\right)=min\left[1,max(0,\rho_{th,k}^{*})\right]$$

This constraint ensures consistency with the normalized BDS reliability score and prevents non-physical threshold values.

To suppress frequent switching caused by small fluctuations around a single threshold, two hysteresis thresholds are constructed:

(74) $$\{\begin{array}{c} \rho_{on,k}=\mathrm{clip}\left(\rho_{th,k}+h,0,1\right)\\ \;\;\rho_{off,k}=\mathrm{clip}\left(\rho_{th,k}-h,0,1\right) \end{array}\;\;\;\;\;\;h>0$$

where h is the dimensionless hysteresis half-width. The upper threshold $\rho_{on,k}$ is used when switching from eLoran back to BDS, whereas the lower threshold $\rho_{off,k}$ is used when determining whether the system should remain with BDS. Thus, once the system has switched to eLoran, BDS must recover to a higher reliability level before it is selected again.

Let $s_{k}\in \left\{BDS,LW\right\}$ denote the observation source selected at epoch k, and let $s_{k-1}$ denote the source selected at the preceding epoch. Combining the BDS availability condition, reliability score, normalized-innovation gate, and hysteresis thresholds, the source-selection rule is defined as [29]

(75) $$s_{k}=\{\begin{array}{c} \begin{array}{cc} LW & if\;BDS\;is\;unavailable \end{array}\\ \begin{array}{cc} BDS & if\;s_{k-1}=BDS,\;\rho_{BDS,k}\geq \rho_{off,k},\;\eta_{BDS,k}\leq \eta_{max}\; \end{array}\\ \begin{array}{cc} BDS & if\;s_{k-1}=LW,\;\rho_{BDS,k}\geq \rho_{off,k},\;\eta_{BDS,k}\leq \eta_{max} \end{array}\\ \begin{array}{cc} LW & otherwise \end{array} \end{array}$$

where $\eta_{max}>0$ is the prescribed upper limit of the normalized BDS innovation. The reliability score provides a signal-quality criterion, whereas the normalized-innovation gate prevents a BDS observation that is inconsistent with the predicted state from entering the measurement update, even if its signal-quality indicators appear acceptable.

The sliding-window statistics in Equations (66)–(71) are updated only when the BDS observation is accepted under nominal conditions, namely when its reliability and normalized innovation satisfy the corresponding acceptance criteria. During BDS degradation, outage, or observation-source switching, these reference statistics are held unchanged. This prevents abnormal observations from gradually shifting the nominal reliability and innovation baselines.

After the observation source $s_{k}\;$ has been selected, the measurement used in the Kalman-filter update is

(76) $$z_{k}=\{\begin{array}{c} z_{BDS\;,\;k}\;\\ z_{LW\;,\;k}\;\;\; \end{array}$$

The corresponding measurement matrix is

(77) $$H_{k}=\{\begin{array}{c} \;\;H_{BDS\;,\;k}\;\;,\;\;s_{k}=BDS\;\;\;\\ H_{LW\;,\;k}\;\;\;,\;\;s_{k}=LW\;\; \end{array}$$

and the measurement-noise covariance is

(78) $$R_{k}=\{\begin{array}{c} \;\;R_{BDS\;,\;k}\;\;,\;\;s_{k}=BDS\;\;\;\\ R_{LW\;,\;k}\;\;\;,\;\;s_{k}=LW\;\; \end{array}$$

Thus, the observation, measurement matrix, and measurement-noise covariance are switched consistently as a group. Although Equation (53) provides a joint-measurement form for cases in which both observations are simultaneously used, the source-selection implementation adopted in the present three-layer architecture uses one external observation source for each measurement update.

If neither BDS nor eLoran provides a valid observation at epoch k, the measurement update is skipped and the time output is maintained using the Kalman state prediction. Once a valid external observation becomes available again, the measurement update is resumed using the corresponding measurement model.

The adaptive adjustment of $R_{BDS,k}$, the normalized-innovation gate, and the hysteresis-based source-selection mechanism reduce the influence of degraded or inconsistent BDS observations and suppress frequent switching between BDS and eLoran. Nevertheless, these mechanisms do not establish that the underlying BDS errors are Gaussian, nor do they completely eliminate the effects of abrupt biased or heavy-tailed errors. They should therefore be regarded as adaptive weighting and observation-screening mechanisms within the Kalman-filter framework.

### 4.6. KF Prediction, Update, and Time Output

For the KF, considering the short-term drift of the oscillator and the accumulation of noise, the prediction step is as follows [30]:

(79) $${\hat{x}}_{k|k-1}=F{\hat{x}}_{k-1|k-1}$$

(80) $$P_{k|k-1}=FP_{k-1|k-1}F^{T}+Q_{k-1}$$

where ${\hat{x}}_{k|k-1}$ and ${\hat{x}}_{k-1|k-1}$ denote the predicted state at time k based on information available at time k−1 and the updated state estimate at time k−1, respectively. $P_{k|k-1}$ and $P_{k-1|k-1}$ denote the corresponding predicted covariance matrix and updated covariance matrix, respectively. $Q_{k-1}$ is the process-noise covariance over the prediction interval from epoch k−1 to epoch k, as defined in Section 4.2.

After completing the prediction step, according to Equations (76)–(78), the innovation corresponding to the selected observation source is then calculated as

(81) $$v_{k}=z_{k}-H_{k}{\hat{x}}_{k|k-1}$$

where $v_{k}$ represents the difference between the selected external time observation and the observation predicted from the current state estimate. It contains the correction information provided by the selected BDS or eLoran observation for the local time reference.

The innovation covariance is calculated as

(82) $$S_{k}=H_{k}P_{k|k-1}H_{k}^{T}+R_{k}$$

In the source-selection implementation adopted in this study, only one external observation source is used in each measurement update. Therefore, $z_{k}$, $\nu_{k}$, $S_{k}$, and $R_{k}$ are scalars, whereas $H_{k}$ is a 1×5 measurement matrix.

The Kalman gain is calculated as

(83) $$K_{k}=P_{k|k-1}H^{T}{S_{k}}^{-1}$$

The value of $K_{k}$ depends on both the predicted state uncertainty and the measurement-noise covariance of the selected observation source. When BDS is selected, $R_{k}=R_{BDS,k}$, which is dynamically adjusted according to the BDS reliability score. When eLoran is selected, $R_{k}=R_{LW}$, which is treated as fixed under the nominal conditions of the finite-duration controlled simulation.

The posterior state estimate is updated using the innovation as

(84) $${\hat{x}}_{k|k}={\hat{x}}_{k|k-1}+K_{k}v_{k}$$

To reduce the influence of finite-precision round-off errors on the posterior covariance matrix, the Joseph stabilized form is adopted for the covariance update [31]:

(85) $$P_{k|k}=\left(I-K_{k}H_{k}\right)P_{k|k-1}{\left(I-K_{k}H_{k}\right)}^{T}+K_{k}R_{k}K_{k}^{T}$$

where I is the 5×5 identity matrix corresponding to the five-state model.

Under exact arithmetic and with the optimal Kalman gain, the Joseph form is mathematically equivalent to the simplified covariance update. Under finite-precision computation, however, it reduces the risk that repeated iterations, adaptive changes in $R_{BDS,k}$, or observation-source switching cause the covariance matrix to lose symmetry or positive semi-definiteness, although it requires additional matrix multiplications [32,33]. Therefore, the Joseph form, rather than the simplified covariance-update equation, is used in the present implementation.

To further suppress small numerical asymmetries introduced by floating-point computation, the posterior covariance matrix is symmetrized after the Joseph update:

(86) $$P_{k|k}\leftarrow \frac{1}{2}\left(P_{k|k}+P_{k|k}^{T}\right)$$

This operation corrects only numerical asymmetry and cannot independently restore positive semi-definiteness if negative eigenvalues have already occurred. Therefore, it is used as a supplementary numerical treatment and does not replace the Joseph covariance update [34].

After completing the state prediction and, when available, the measurement update, the fused time output is obtained as

(87) $$t_{out,k}=t_{XO,k}-{\hat{\delta t}}_{k}^{+}$$

where ${\hat{\delta t}}_{k}^{+}$ is the updated estimate of the time phase error $\delta t_{k}$ obtained by the Kalman filter at the k-th time instant. $t_{XO,k}$ denotes the local time output directly generated by the temperature-compensated crystal oscillator (TCXO) at the k-th sampling instant, and $t_{out,k}$ denotes the final time-reference output corrected through multi-source time fusion and Kalman filtering.

After KF-based state prediction and measurement correction, $t_{out,k}$ can be directly used as the time reference of the chip system. Meanwhile, the posterior estimates of $y_{k}$ and ${\dot{y}}_{k}$ provide the initial frequency states for the next prediction cycle. The BDS and eLoran bias-state estimates are also propagated to the next epoch according to their respective first-order Gauss–Markov models [2]. In addition, the accepted BDS innovation is supplied to the gated residual-feedback module described in Section 4.6.

### 4.7. KF Residual Closed-Loop Feedback

The digital temperature-compensation module described in Section 2 provides the compensated TCXO frequency sequence used to generate the local time output. As defined in Section 4.1, the Kalman-filter state $y_{k}$ represents the residual fractional-frequency offset after digital temperature compensation. Therefore, the temperature-compensation coefficients are not introduced as additional Kalman-filter states. Instead, the BDS innovation obtained from the Kalman filter is conditionally fed back to the external temperature-compensation module to update its coefficients. The updated coefficients act on the subsequent compensated-frequency sequence and are consequently reflected in the evolution of $y_{k}$.

To ensure dimensional consistency in the feedback update, the temperature variables are first normalized to eliminate the dimensional mismatch between the first- and second-order temperature terms. Meanwhile, the first difference of the time-domain innovations at two consecutive epochs is converted into an equivalent fractional-frequency residual. The dimensionless temperature deviation and temperature-feature vector are defined as follows [14]:

(88) $$u_{k}=\frac{\Delta T_{k}}{T_{s}}$$

(89) $$\psi_{T,k}=\left[\begin{array}{c} u_{k}\\ u_{k}^{2} \end{array}\right]$$

where $T_{s}$ is the temperature-normalization scale, which can be determined according to the calibration temperature range or the typical range of temperature variations. Accordingly, the temperature-compensation parameter vector is defined as

(90) $${\tilde{\theta }}_{k}=\left[\begin{array}{c} {\tilde{\alpha }}_{k}\\ {\tilde{\beta }}_{k} \end{array}\right]$$

(91) $${\tilde{\alpha }}_{k}={\hat{\alpha }}_{k}T_{s},{\tilde{\beta }}_{k}={\hat{\beta }}_{k}T_{s}^{2}$$

where ${\hat{\alpha }}_{k}$ and ${\hat{\beta }}_{k}$ are the estimates of the first- and second-order temperature-compensation coefficients, α and β, respectively, at epoch k. Since $\theta_{k}^{T}\psi_{T,k}$ represents the temperature-induced fractional-frequency offset calculated using the current temperature-compensation parameters, the compensation term can be expressed as

(92) $${\tilde{\theta }}_{k}^{T}\psi_{T,k}={\hat{\alpha }}_{k}\Delta T_{k}+{\hat{\beta }}_{k}\Delta T_{k}^{2}$$

The fractional-frequency offset after digital temperature compensation is therefore given by

(93) $$y_{comp,k}=y_{raw,k}-{\tilde{\theta }}_{k}^{T}\psi_{T,k}$$

where $y_{raw,k}$ and $y_{comp,k}$ denote the TCXO fractional-frequency offsets before and after digital temperature compensation, respectively. The compensated sequence $y_{comp,k}$ is used to generate the local oscillator time $t_{XO,k}$ and provides the frequency sequence represented by the Kalman-filter state $y_{k}$.

The Kalman innovation $\nu_{k}$ defined in Equation (81) has units of time. However, only innovations obtained from consecutively accepted BDS observations are used for temperature-compensation feedback. The eLoran innovation is not used for this purpose because its lower update rate and propagation-related bias variation may be transferred into the temperature-compensation parameters. When BDS is selected at two consecutive epochs, the first difference of the corresponding BDS innovations is divided by the filtering interval to construct an equivalent residual fractional-frequency signal [9]:

(94) $$e_{y,k}=\frac{v_{BDS,k}-v_{BDS,k-1}}{\Delta t}$$

where Δt is the time interval between two consecutive filtering epochs. 

The quantity $e_{y,k}$ should be regarded as a feedback proxy for the residual fractional-frequency error rather than as an independent direct measurement of the TCXO frequency error. In addition to the residual oscillator-frequency component, the innovation difference may contain changes in the BDS link bias, short-term BDS measurement noise, and state-prediction errors. Under reliable BDS conditions and over a short filtering interval, $b_{BDS,k}$ is assumed to vary slowly, so differencing suppresses its approximately common component between two adjacent epochs. Nevertheless, it cannot completely eliminate link-bias variations or measurement noise. Therefore, smoothing and feedback gating are required before $e_{y,k}$ is used to update the temperature-compensation parameters.

To prevent unreliable observations and observation-source switching from entering the feedback loop, the feedback-gating coefficient $g_{k}$ is defined as

(95) $$g_{k}=\{\begin{array}{c} \begin{array}{cc} 1 & \begin{array}{c} s_{k}=s_{k-1}=BDS\\ \rho_{BDS,k}\geq \rho_{off,k}\\ \begin{array}{c} \rho_{BDS,k-1}\geq \rho_{off,k-1}\\ \eta_{BDS,k}\leq \eta_{max}\\ \eta_{BDS,k-1}\leq \eta_{max} \end{array} \end{array} \end{array}\\ \begin{array}{cc} 0 & otherwise \end{array} \end{array}$$

Thus, feedback is enabled only when BDS has been continuously selected for two adjacent epochs and both BDS observations satisfy the reliability and normalized-innovation criteria. In particular, $g_{k}=0$ during BDS degradation, BDS outage, eLoran operation, or observation-source switching.

Because the first difference in Equation (94) may amplify short-term measurement noise, first-order smoothing is applied when the feedback gate is open. When the gate is closed, the smoothed residual is held at its previous value:

(96) $$g_{k}=\{\begin{array}{c} \begin{array}{cc} \lambda_{e}{\tilde{e}}_{y,k-1}+\left(1-\lambda_{e}\right)e_{y,k} & g_{k}=1 \end{array}\\ \begin{array}{cc} {\tilde{e}}_{y,k-1} & g_{k}=0 \end{array} \end{array}\;\;\;\;\;0\leq \lambda_{e}\leq 1$$

where $\lambda_{e}$ is the smoothing coefficient. A larger value provides stronger suppression of short-term fluctuations but slows the response of the feedback loop, whereas a smaller value increases the response speed but also increases sensitivity to measurement noise.

Following the normalized least-mean-square parameter-update method [35], the temperature-compensation parameters are corrected in closed loop as follows:

(97) $${\tilde{\theta }}_{k+1}={\tilde{\theta }}_{k}+g_{k}\mu \frac{{\bar{e}}_{y,k}\psi_{T,k}}{\psi_{T,k}^{T}\psi_{T,k}+\varepsilon_{T}}$$

where μ is the dimensionless feedback step size, $\varepsilon_{T}>0$ is a small regularization constant introduced to prevent division by zero, and $g_{k}\in [0,1]$ is the feedback-gating coefficient. Because the temperature-feature vector has been normalized, both $\psi_{T,k}^{T}\psi_{T,k}$ and $\varepsilon_{T}$ are dimensionless.

When $g_{k}=0$, Equation (97) gives ${\tilde{\theta }}_{k+1}\approx {\tilde{\theta }}_{k}$, so the temperature-compensation coefficients are frozen. When $g_{k}=1$, the parameter correction calculated at epoch k is applied to the compensation of the subsequent frequency sample. This causal update order avoids directly using the current innovation to modify the frequency sample that has already been used in the same Kalman-filter cycle.

To prevent long-term parameter drift caused by accumulated residual noise, the feedback step size μ should be selected conservatively, and physically reasonable bounds may be imposed on ${\hat{\alpha }}_{k}$ and ${\hat{\beta }}_{k}$ according to the TCXO calibration results. In the controlled simulation, the values of $T_{s}$, $\lambda_{e}$, μ, and $\varepsilon_{T}$, together with the initial values of the temperature-compensation coefficients, should be explicitly reported to ensure reproducibility.

The residual-feedback loop should be regarded as a conditionally activated enhancement rather than an always-beneficial component of the time-fusion method. It is enabled only under stable and reliable BDS observations. The gate is closed under degraded observations, eLoran backup operation, or observation-source switching to prevent source-specific link errors and switching discontinuities from being absorbed into the temperature-compensation coefficients. This design reduces the risk of overcompensation but does not completely eliminate the possibility that unmodeled BDS bias variations or residual measurement noise affect the feedback update.

## 5. Results and Discussion

### 5.1. Simulation Design and Evaluation Metrics

To evaluate the proposed three-layer time-fusion architecture and the reformulated five-state Kalman filter, a 24-h controlled simulation was conducted with a basic filtering interval of 1 s. The simulation incorporates temperature-dependent TCXO frequency variations, changes in BDS observation quality, BDS degradation and outage, and eLoran backup observations, thereby allowing the timing performance to be examined over different time scales and observation conditions.

The BDS signal-quality information was obtained from an actual RINEX observation file with an original sampling interval of 30 s. Quality indicators, including the carrier-to-noise density ratio, the number of usable BDS satellites, and the code-rate median absolute deviation, were extracted and resampled to 1 s for calculating the comprehensive BDS reliability score and dynamically adjusting the observation-noise covariance. It should be emphasized that the resampling was applied only to the signal-quality indicators, rather than directly to the original pseudo-range or carrier-phase observations. The continuous 1-s timing output between adjacent original observation epochs was maintained by the state prediction of the Kalman filter.

The simulated TCXO includes temperature-dependent frequency variation, an initial frequency offset, aging, white frequency noise, and random-walk frequency noise. The reference temperature is 25 °C, and the linear and quadratic temperature coefficients are $8.0\times 10^{-10}$/°C and $2.5\times 10^{-10}$/°C^2^, respectively. The initial fractional-frequency offset and aging rate are $1.0\times 10^{-9}$ and $5.0\times 10^{-15}$/s, while the white-noise and random-walk-noise standard deviations are $2.0\times 10^{-11}$ and $5.0\times 10^{-15}$ per 1-s step, respectively.

The 24-h experiment was divided into five stages: normal BDS observations from 0 to 6 h, BDS degradation from 6 to 8 h, recovery from 8 to 12 h, a controlled BDS outage from 12 to 16 h, and post-outage recovery from 16 to 24 h. The controlled outage was implemented using an independent availability mask rather than by artificially modifying the original RINEX quality indicators. This design separates the signal-quality variations contained in the actual record from the observation availability specified by the controlled experiment.

Six schemes were considered for comparison. Exp1 uses the uncompensated TCXO; Exp2 uses the TCXO with fixed digital temperature compensation; Exp3 applies Kalman filtering with a fixed BDS observation-noise covariance; Exp4 applies adaptive Kalman filtering in which the BDS observation-noise covariance is dynamically adjusted according to the BDS reliability score; Exp5a represents TCXO/BDS/eLoran fusion without residual feedback; and Exp5b represents the complete fusion scheme with conditionally gated residual feedback.

The evaluated quantities include output-time error, maximum error, maximum outage error, observation innovations, state-estimation error, and Allan deviation. Two concepts are explicitly distinguished in the analysis: whether an independent external timing constraint remains available during the BDS outage, and whether this constraint actually improves the timing accuracy. The former reflects the external observability and redundancy of the system, whereas the latter also depends on the modeling and calibration of eLoran propagation biases and on the observation-noise level.

### 5.2. Characteristics of the Timing Sources and Adaptive Observation Modeling

**Figure 2:**
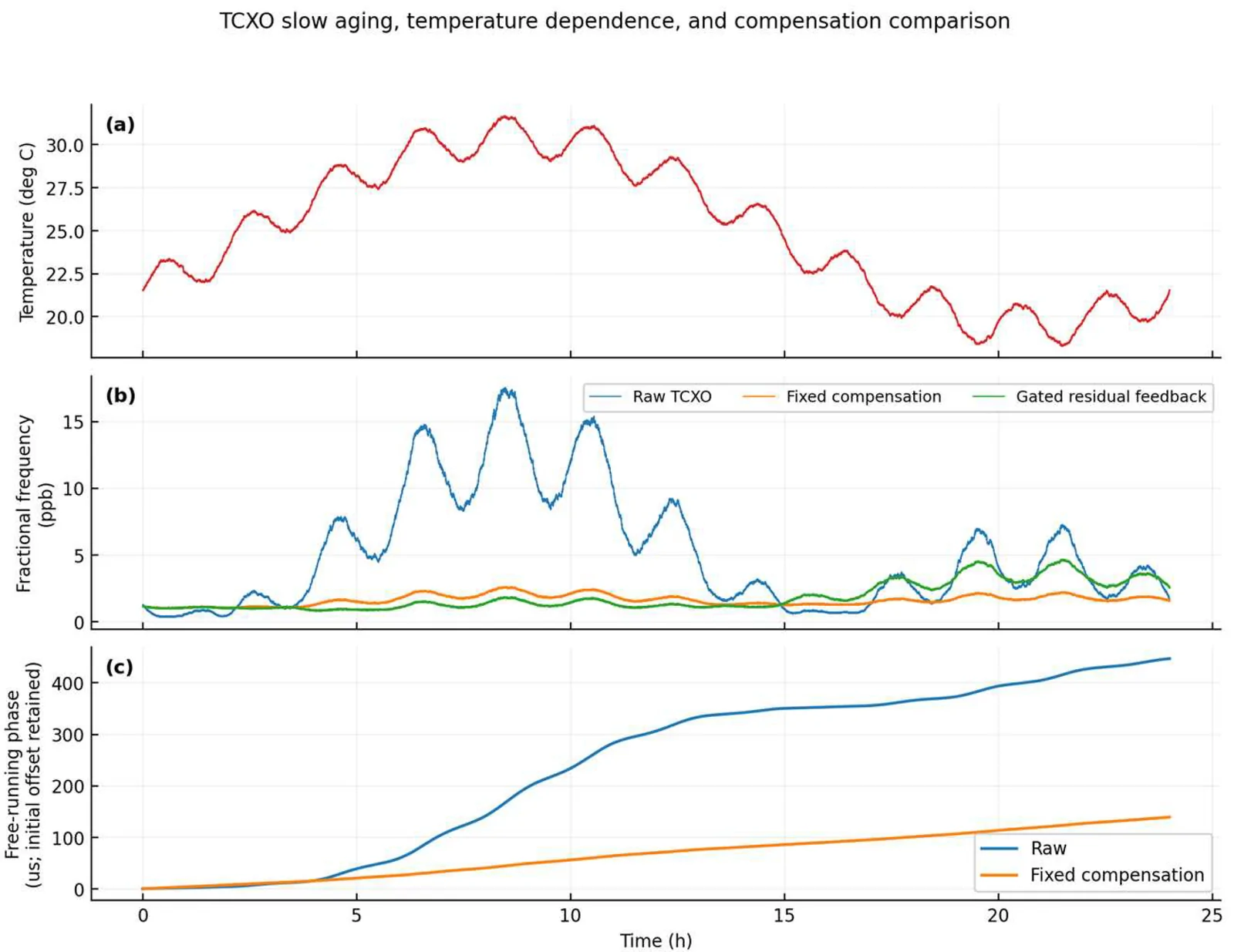
Temperature-dependent characteristics and compensation results of the TCXO over 24 h: (**a**) simulated ambient temperature; (**b**) residual fractional-frequency offsets of the raw TCXO, the fixed digitally temperature-compensated TCXO, and the gated residual-feedback-corrected TCXO; (**c**) free-running time-phase errors of the raw and fixed digitally temperature-compensated TCXOs.

Figure 2 presents the temperature-dependent behavior and compensation results of the TCXO over the 24-h simulation. Figure 2a–c show the ambient temperature, fractional-frequency offsets, and accumulated free-running phase errors, respectively. The gated residual-feedback result is included only in Figure 2b, because it represents a closed-loop correction rather than a free-running oscillator output.

As shown in Figure 2a, the simulated temperature varies from approximately 18.3 to 31.6 °C and contains a slowly varying diurnal trend, local periodic fluctuations, and small random perturbations. This profile represents the non-isothermal operating condition, while aging and random frequency noise are introduced separately into the simulated TCXO frequency sequence.

In Figure 2b, the raw TCXO frequency offset varies from approximately 0.33 to 17.48 ppb and is clearly correlated with temperature. After applying the fixed digital temperature-compensation model defined in Equation (14), the offset is reduced to approximately 0.96–2.59 ppb. The remaining low-frequency offsets and random fluctuations indicate that fixed temperature compensation cannot completely eliminate calibration errors, aging, or stochastic oscillator noise.

Gated residual feedback further reduces the frequency residual during some intervals but does not consistently outperform fixed temperature compensation. Its effectiveness depends on the innovation, BDS reliability, and stability of the active observation source. It is therefore activated only when the gating conditions are satisfied and is treated as a conditional rather than universally beneficial correction.

As shown in Figure 2c, residual frequency offsets accumulate through temporal integration. At the end of 24 h, the free-running phase error is approximately 447 μs for the raw TCXO and 139 μs after fixed digital temperature compensation, corresponding to a reduction of about 69%. Nevertheless, the compensated error continues to increase, indicating that the TCXO provides a continuous short-term local reference but still requires external BDS or eLoran constraints for medium- and long-term correction.

**Figure 3:**
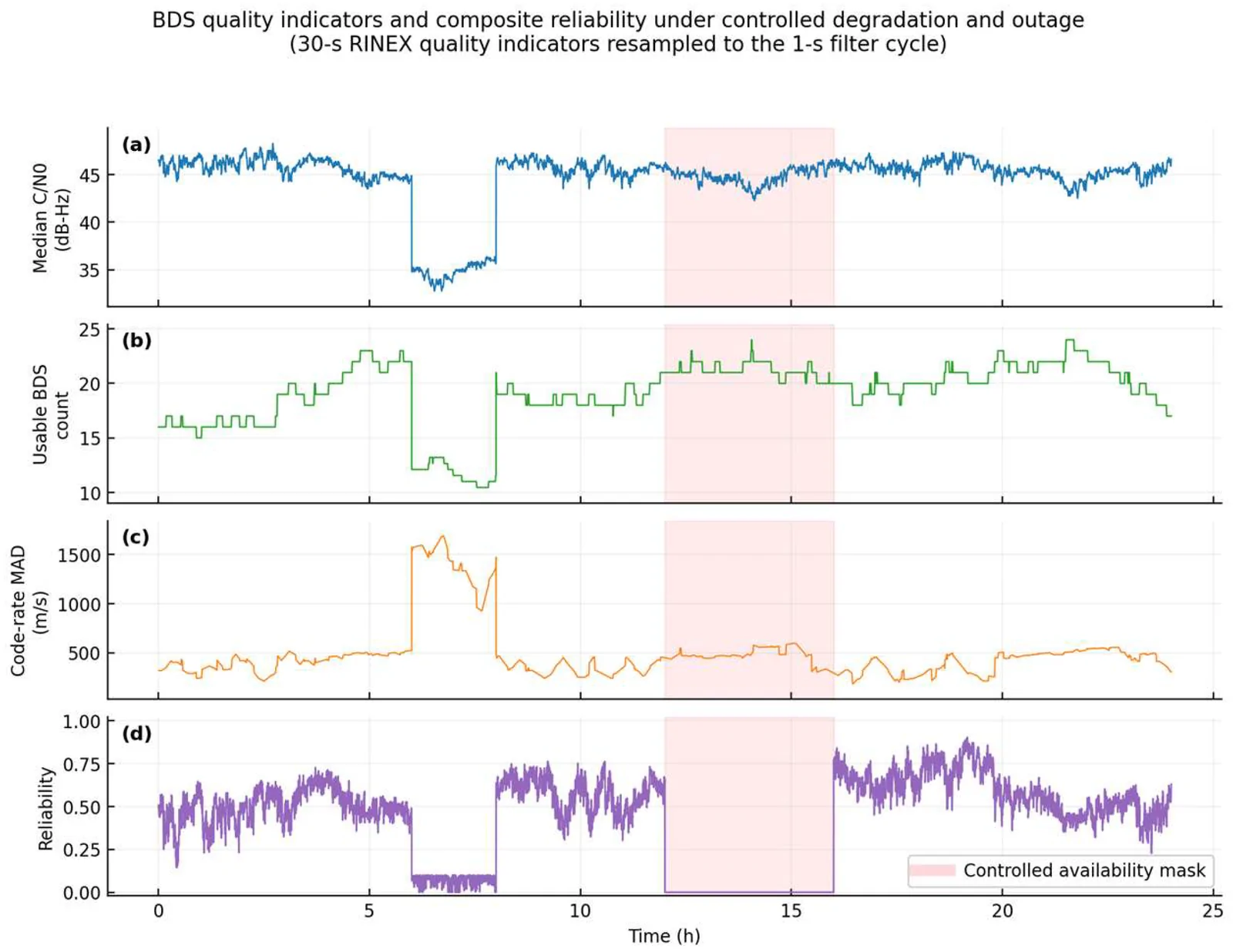
BDS quality indicators constructed from the 30-s RINEX observation file and the composite reliability under the controlled scenario: (**a**) median carrier-to-noise density ratio; (**b**) number of usable BDS satellites; (**c**) code-rate median absolute deviation; (**d**) composite BDS reliability score and controlled availability mask. The shaded region denotes the controlled BDS-unavailable interval from 12 to 16 h.

Figure 3 presents the BDS quality indicators extracted from the original 30-s RINEX file and resampled to the 1-s filter cycle, together with the resulting reliability score. Resampling is applied only to the quality indicators used for reliability assessment, not to the original pseudo-range or carrier-phase observations. Continuous output between adjacent RINEX epochs is maintained by Kalman-filter state prediction rather than observation interpolation.

During normal and recovery intervals, the indicators retain the variations contained in the RINEX record. Controlled degradation is introduced from 6 to 8 h by reducing the carrier-to-noise density ratio and usable-satellite count and increasing the geometric and code-rate dispersion indicators. From 12 to 16 h, BDS is declared unavailable through an independent availability mask, while the underlying RINEX quality indicators are retained. This treatment distinguishes degraded signal quality from complete source unavailability.

The composite reliability score decreases during degradation, is set to zero during the controlled outage, and returns to its normal range after recovery. According to Equation (22), a lower score increases the BDS observation-noise covariance and reduces the weight of degraded observations in the Kalman update. This adaptive weighting improves robustness but does not constitute evidence that the BDS residuals are Gaussian.

**Figure 4:**
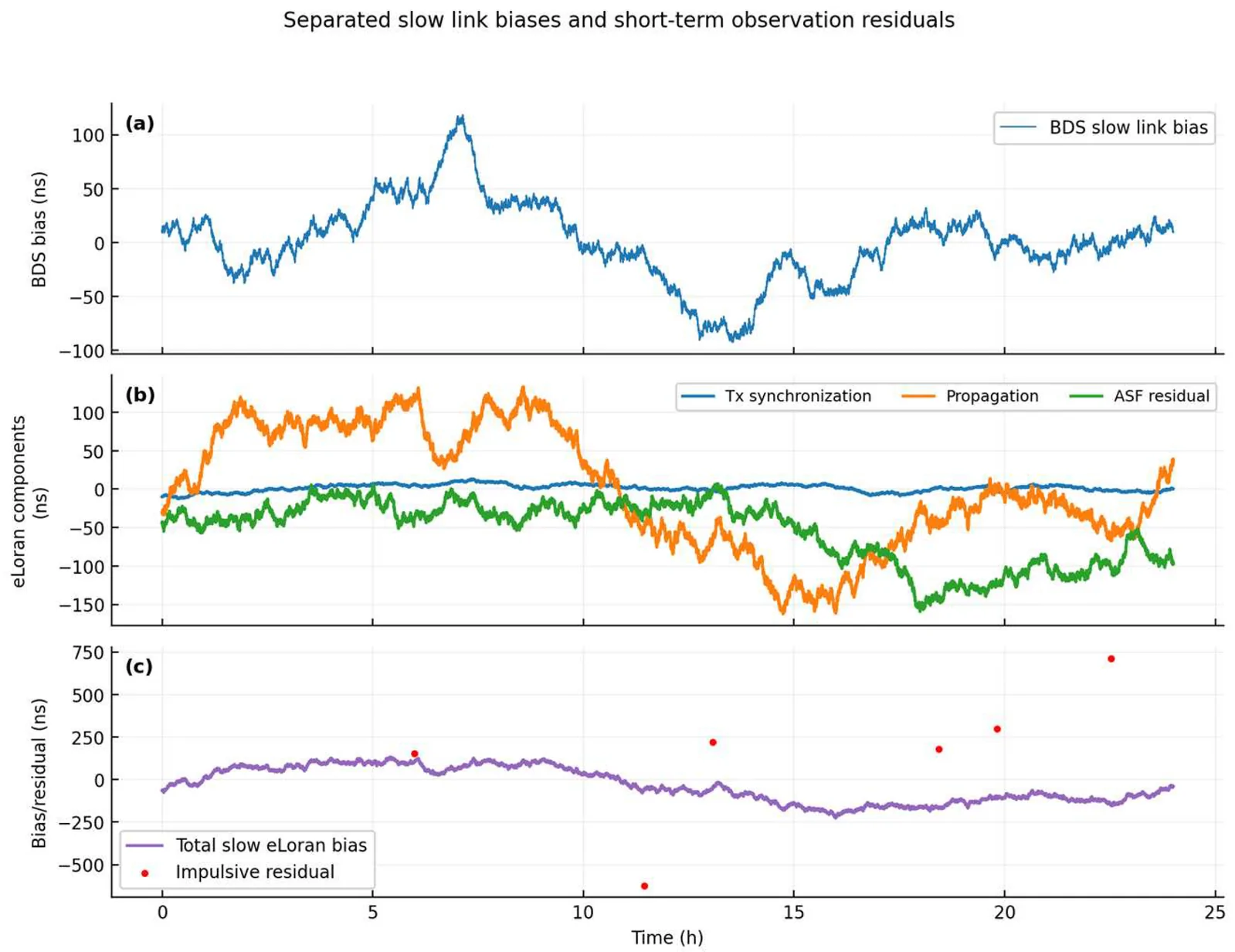
Separation of slowly varying link biases and short-term observation residuals in the 24-h controlled simulation: (**a**) equivalent slowly varying BDS link bias; (**b**) eLoran transmitter-synchronization, propagation-delay, and ASF-residual components; (**c**) total slowly varying eLoran link bias and representative impulsive residuals.

Figure 4 illustrates the separation between slowly varying link biases and short-term residuals in the revised BDS and eLoran observation models. It describes the construction and physical boundaries of the simulated error components rather than empirically validating the probability distributions of actual observations.

The BDS link-bias state $b_{BDS,k}$ in Figure 4a ranges from approximately −92 to 117 ns and represents the combined effects of incompletely corrected atmospheric and hardware delays and persistent multipath or NLOS biases. Faster observation fluctuations are assigned to the short-term residual $\varepsilon_{BDS,k}$, avoiding duplicate representation of persistent propagation errors in both the bias state and observation noise.

As shown in Figure 4b, the eLoran transmitter-synchronization, propagation-delay, and ASF-residual components vary slowly and are incorporated into $b_{LW,k}$. The discrete impulses in Figure 4c represent possible atmospheric impulsive noise, abnormal TOA estimates, or cycle-selection errors. Only after separating the slowly varying bias and rejecting evident outliers is the remaining short-term residual conditionally approximated as zero-mean Gaussian under the controlled nominal conditions.

These results confirm the complementary roles of the three timing sources. The TCXO provides continuous short-term output, BDS supplies quality-dependent external observations, and eLoran maintains an independent external constraint when BDS is degraded or unavailable. However, eLoran timing accuracy remains dependent on propagation-delay and ASF calibration; therefore, maintaining external observability does not necessarily guarantee a reduction in the maximum outage error.

### 5.3. Time-Domain Error Comparison of Different Fusion Methods

**Figure 5:**
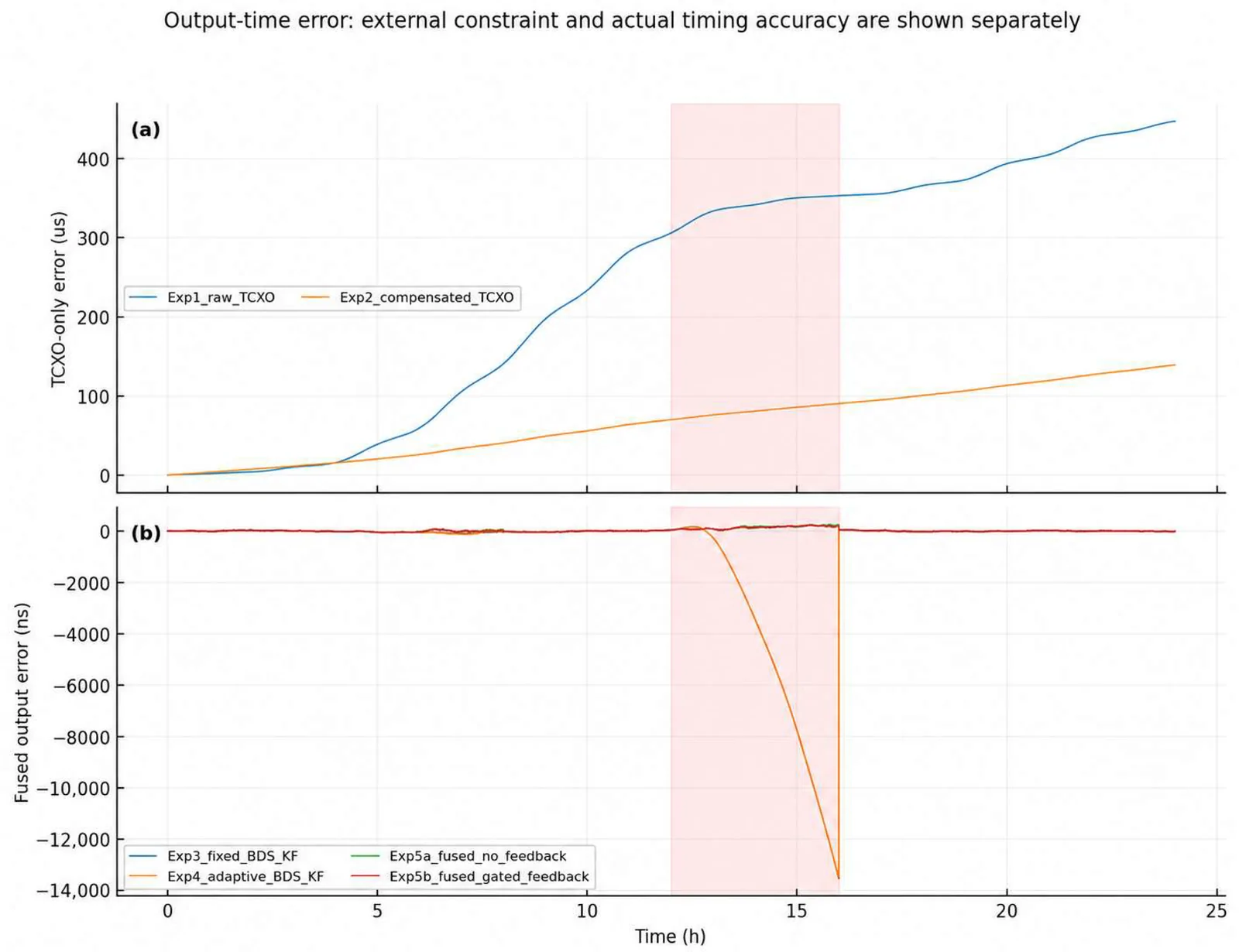
Time-domain error comparison of different timing and fusion methods over the 24-h controlled simulation: (**a**) free-running time errors of the raw TCXO and the fixed digitally temperature-compensated TCXO; (**b**) output time errors of the fixed BDS-KF, adaptive BDS-KF, three-layer fusion method without feedback, and three-layer fusion method with gated residual feedback. The shaded region denotes the controlled BDS-unavailable interval from 12 to 16 h.

Figure 5 compares the output time errors of the six methods over the 24-h controlled simulation. Because the accumulated errors of the TCXO-only methods are substantially larger than those of the Kalman filter-based methods, Figure 4a presents the raw and fixed digitally temperature-compensated TCXO results in μs, whereas Figure 4b compares the four Kalman filter-based methods in ns. The shaded region denotes the controlled BDS-unavailable interval from 12 to 16 h.

As shown in Figure 5a, the time error of the raw TCXO continuously accumulates because of its residual frequency offset and reaches approximately 447 μs at the end of 24 h. Fixed digital temperature compensation reduces this error to approximately 139 μs, corresponding to a reduction of about 69% in the accumulated error. Nevertheless, the compensated time error continues to increase, indicating that temperature compensation alone cannot constrain the long-term integration of the residual frequency offset.

During the normal and recovery stages of BDS operation, all four Kalman filter-based methods use the external BDS observations to constrain the local-clock error. Within the controlled degradation interval from 6 to 8 h, the fixed BDS-KF retains a constant observation-noise covariance, whereas the adaptive BDS-KF reduces the weight of degraded observations according to the BDS reliability score. The adaptive mechanism therefore mainly limits the instantaneous influence of degraded observations and cannot replace the unavailable external measurements during a complete outage. Because the errors accumulated during the 12–16 h BDS outage dominate the overall 24-h metrics, the full-period RMSE values of the fixed and adaptive BDS-KF methods are similar, at 2484.51 and 2476.44 ns, respectively.

During the BDS-unavailable interval from 12 to 16 h, the fixed and adaptive BDS-KF methods rely only on TCXO state prediction to maintain continuous output, and their maximum outage errors increase to 13.52 and 13.47 μs, respectively. In contrast, Exp5a and Exp5b accept the backup eLoran observations at a 30-s update interval, thereby retaining an independent external timing constraint and limiting the maximum outage errors to 254.47 and 255.32 ns, respectively. Under the nominal propagation-error settings adopted in this experiment, eLoran therefore improves both external observability and actual outage timing accuracy. This result, however, depends on the modeling and calibration of the propagation-delay and ASF biases and should not be interpreted as evidence that eLoran always outperforms TCXO holdover under arbitrary propagation conditions.

In this 24-h realization, Exp5b with gated residual feedback achieves a lower RMSE and maximum source-switching jump than Exp5a without feedback, whereas its maximum absolute error and maximum outage error are slightly higher. Specifically, the RMSE decreases from approximately 68.9 to 63.7 ns, and the maximum source-switching jump decreases from approximately 179 to 125 ns, whereas the maximum absolute error and maximum outage error change from approximately 254.47 to 255.32 ns. This result indicates that residual feedback can suppress part of the local drift and source-switching disturbance when the innovation, BDS reliability, and observation-source stability satisfy the gating conditions, but it does not consistently improve all timing metrics. Nevertheless, a single simulation realization does not demonstrate that the feedback mechanism is beneficial under all conditions, and its overall effectiveness must also be evaluated using the statistical results of repeated random trials.

Overall, the three-layer fusion architecture maintains a continuous time output under degraded or unavailable BDS conditions and preserves an external timing constraint through eLoran. However, maintaining external observability, improving output continuity, and improving actual timing accuracy are distinct performance objectives; the latter two remain dependent on the eLoran propagation biases, observation noise, and residual-feedback gating conditions.

### 5.4. Internal Variables and Source-Switching Mechanism of the Complete Method

**Figure 6:**
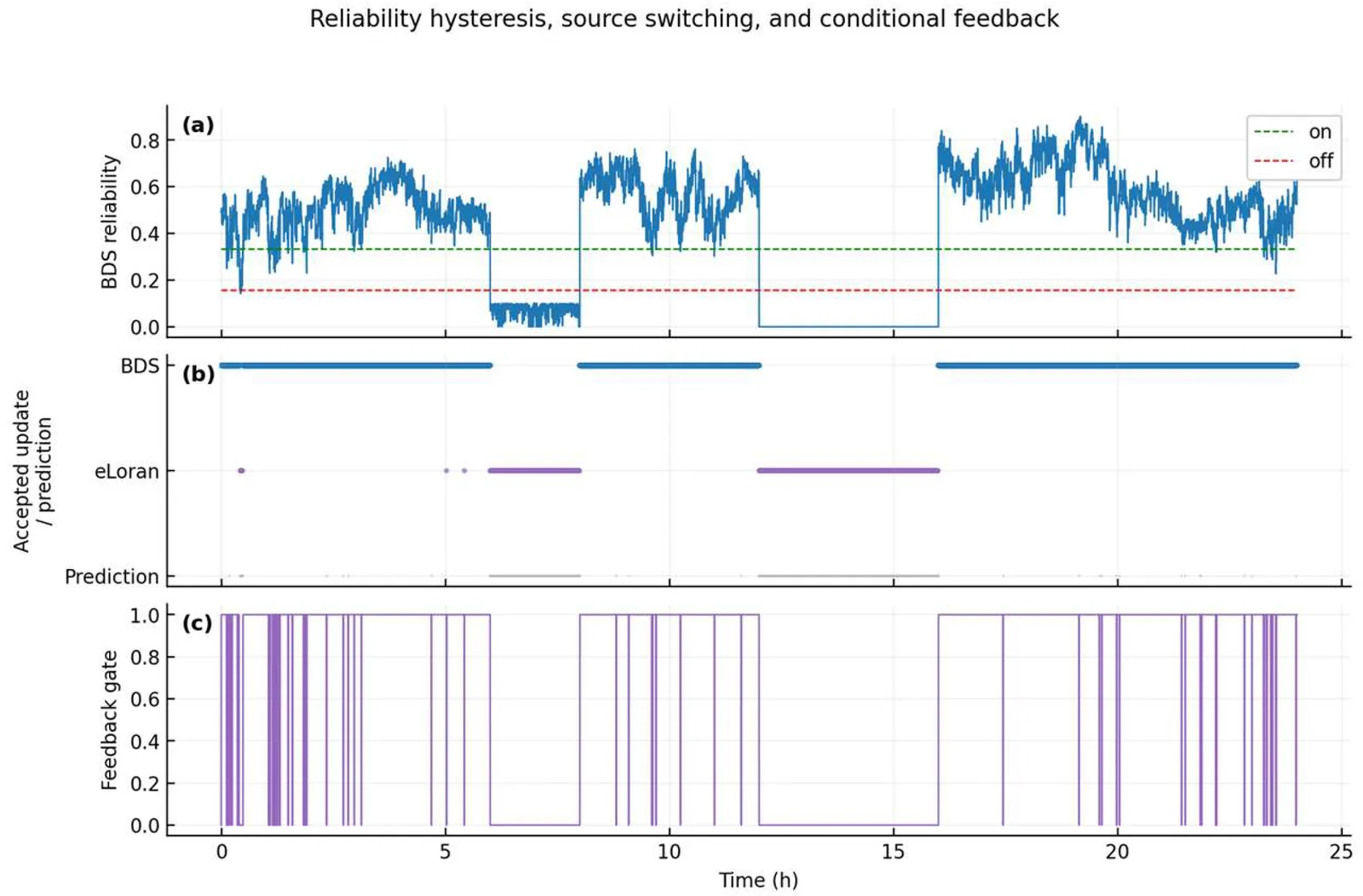
Reliability assessment, observation-source switching, and conditional feedback of the complete three-layer fusion method: (**a**) composite BDS reliability score and hysteretic switching thresholds; (**b**) accepted BDS or eLoran updates and prediction-only epochs; (**c**) residual-feedback gating state.

**Figure 7:**
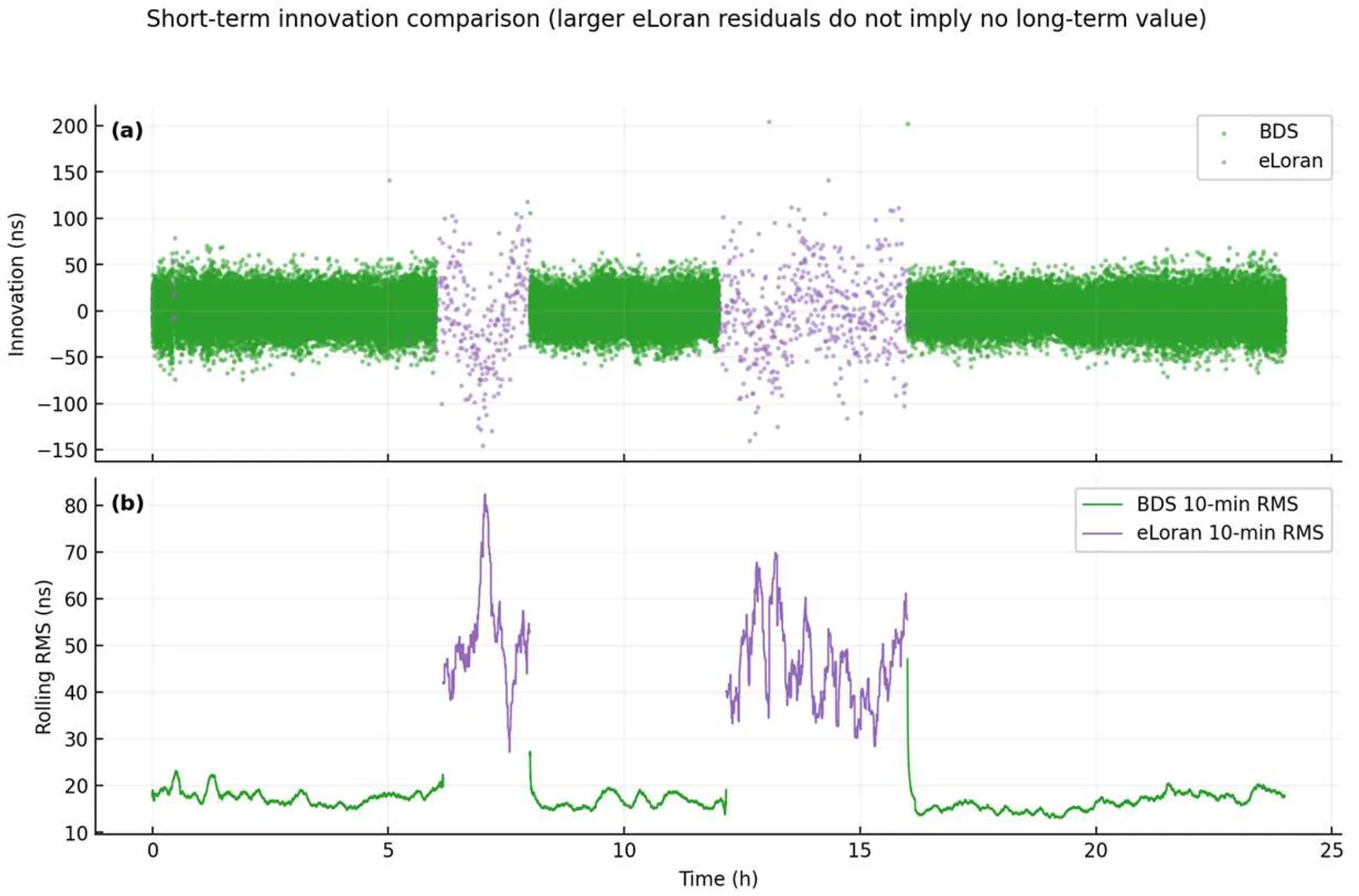
Short-term innovation characteristics of BDS and eLoran: (**a**) innovation sequences corresponding to valid measurement updates; (**b**) 10-min rolling root-mean-square values of the BDS and eLoran innovations. Each curve is shown only over intervals in which the corresponding source provides valid updates.

Figure 6 presents the BDS reliability assessment, observation-source selection, and feedback-gating state of the complete three-layer fusion method over the 24-h simulation. Figure 7 further compares the short-term innovations of BDS and eLoran and their 10-min rolling root-mean-square values, illustrating the necessity of conditionally gating the feedback mechanism.

As shown in Figure 6a, the BDS reliability score varies with the signal-quality indicators extracted from the RINEX record. Turn-on and turn-off thresholds of approximately 0.33 and 0.15 form a hysteresis interval. BDS is preferred when the reliability exceeds the turn-on threshold and is disabled when it falls below the turn-off threshold. The controlled degradation from 6 to 8 h markedly reduces the reliability score, whereas the independent availability mask sets it to zero from 12 to 16 h. This hysteretic decision rule prevents frequent back-and-forth switching when the reliability fluctuates near a single threshold.

Figure 6b shows that the filter primarily accepts BDS updates during normal and recovery intervals in which BDS is reliable. During the controlled degradation and unavailable intervals, eLoran backup observations are accepted at a 30-s interval, while continuous output between adjacent eLoran epochs is maintained through 1-s state prediction. The “Prediction” points therefore represent the normal prediction process between valid external updates rather than a prolonged interval of unconstrained pure holdover. Occasional eLoran updates or prediction-only epochs outside the controlled intervals result from variations in the measured reliability and innovation gating.

Observation-source switching is not determined solely from the instantaneous estimates of the link-bias states. As discussed in the identifiability analysis in Section 4, when only one external source is active, the clock-phase error and its source-specific link bias cannot be algebraically separated from a single measurement. Their separation additionally relies on the local-clock dynamic model, stochastic priors assigned to the link biases, initial covariance information, and observations accumulated over multiple epochs. The switching decision therefore uses directly available signal reliability, innovation magnitude, and source stability rather than assuming that both link biases are independently observable at every epoch.

Figure 6c shows that residual feedback is not a continuously active fixed closed-loop correction but is jointly controlled by the BDS reliability, normalized innovation, and duration of observation-source stability. Feedback is disabled for most of the controlled degradation, BDS-unavailable, and source-switching intervals to prevent abnormal innovations or inter-source offsets from entering the temperature-compensation loop. In the present 24-h experiment, feedback is enabled for only approximately 30.0% of the epochs, consistent with its role as a conditional enhancement rather than an always-beneficial correction component.

As shown in Figure 7, the BDS innovations during normal and recovery intervals are mainly concentrated around zero, with a 10-min rolling RMS generally ranging from approximately 14 to 22 ns. The eLoran innovations exhibit greater dispersion, with rolling RMS values of approximately 30–80 ns over their active intervals and a small number of anomalous components exceeding 100 ns. This behavior is consistent with the revised observation model: eLoran provides an independent long-term external constraint, but its short-term residuals are affected by TOA-estimation noise and impulsive anomalies and are generally larger than the BDS residuals under nominal conditions.

The larger short-term eLoran innovations do not imply an absence of long-term backup value, but they show that the complete eLoran residual should not be directly fed back to the local oscillator. A residual is used for closed-loop frequency correction only when the innovation passes the gating test, the selected observation source remains stable, and the reliability conditions are satisfied. Otherwise, the filter uses only the covariance-weighted measurement update or retains state prediction. This mechanism reduces the risk of erroneous feedback caused by source-switching transients, abnormal TOA estimates, or impulsive noise.

These results show that observation-source switching and residual feedback serve different functions. Source switching preserves an external timing constraint when BDS is degraded or unavailable, whereas residual feedback corrects the remaining post-compensation frequency offset only when the observation conditions are reliable and stable. This design improves robustness against BDS degradation and outage but does not guarantee that residual feedback improves every simulation realization or every timing metric.

### 5.5. Quantitative Metrics and Allan-Deviation Analysis

**Figure 8:**
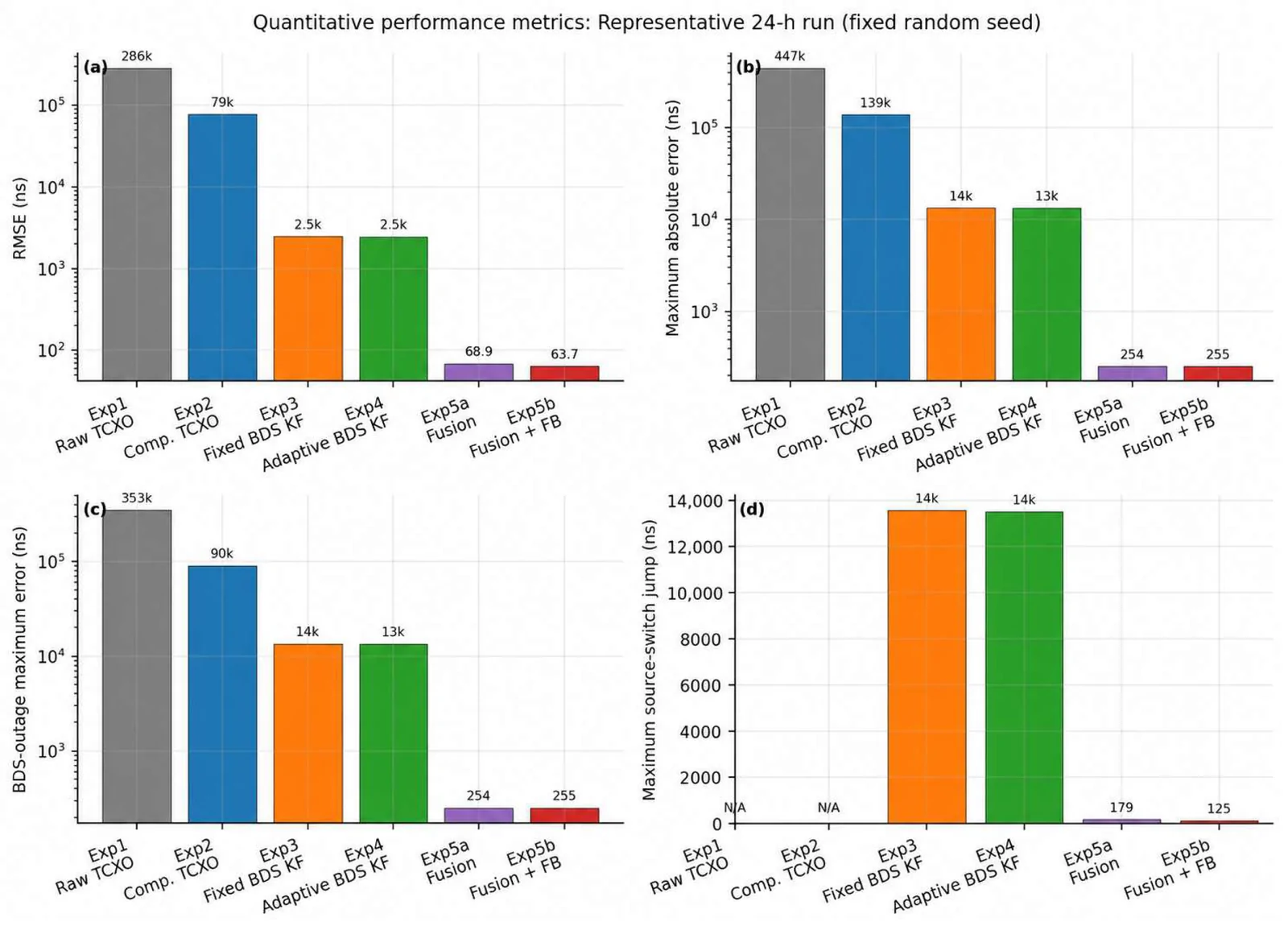
Quantitative performance metrics calculated from the representative 24-h controlled simulation with a fixed random seed: (**a**) overall root-mean-square error; (**b**) maximum absolute error; (**c**) maximum absolute error during the controlled BDS outage; (**d**) maximum one-step output jump associated with observation-update-mode switching. The TCXO-only methods do not perform observation-source switching; therefore, their values in (**d**) are not applicable.

Figure 8 summarizes the quantitative performance metrics obtained from the representative 24-h controlled simulation with a fixed random seed. Figure 8a–c present the overall root-mean-square error, maximum absolute error, and maximum absolute error during the controlled BDS outage, respectively. Figure 8d shows the maximum one-step output jump associated with a change in the observation-update mode. Because Exp1 and Exp2 do not perform observation-source switching, the corresponding values in Figure 8d are not applicable.

These results are obtained from the same representative 24-h controlled simulation and should not be interpreted as Monte Carlo means.

As shown in Figure 8a,b, the raw TCXO exhibits an RMSE of approximately 2.86 × 10^5^ ns and a maximum absolute error of approximately 4.47 × 10^5^ ns. Digital temperature compensation reduces these values to approximately 7.9 × 10^4^ ns and 1.39 × 10^5^ ns, corresponding to reductions of approximately 72% and 69%, respectively. However, the remaining errors are still substantially larger than those of the externally constrained methods, confirming that temperature compensation suppresses the dominant temperature-dependent frequency component but cannot independently prevent the long-term accumulation of residual frequency offsets.

The fixed and adaptive BDS Kalman filters both reduce the overall RMSE to approximately $2.5\times 10^{3}$ ns. The adaptive method decreases the maximum absolute error from approximately $1.4\times 10^{4}$ to $1.3\times 10^{4}$ ns by reducing the weight assigned to degraded BDS observations. Nevertheless, the two BDS-only methods exhibit similar errors during the complete BDS outage because neither method receives an external observation update during this interval. Their outage errors are therefore dominated by TCXO state prediction rather than by the difference between fixed and adaptive BDS weighting.

When eLoran is introduced as a backup observation source, Exp5a and Exp5b reduce the overall RMSE to 68.9 and 63.7 ns, respectively. Their maximum BDS-outage errors are 254 and 255 ns, substantially lower than the approximately 13–14 μs obtained by the two BDS-only filters. Thus, under the propagation-bias and calibration settings adopted in the controlled simulation, eLoran not only maintains an external timing constraint but also limits the accumulated outage error. This accuracy improvement is model-dependent and should not be generalized to uncalibrated eLoran operation, because actual performance remains sensitive to transmitter synchronization, propagation-delay calibration, ASF correction, TOA estimation, and time-correlated link biases.

The comparison between Exp5a and Exp5b further confirms that residual feedback is a conditional enhancement. Gated feedback reduces the RMSE from 68.9 to 63.7 ns and the maximum switching jump from 179 to 125 ns, corresponding to improvements of approximately 7.5% and 30%, respectively. However, the maximum absolute error and the BDS-outage maximum error increase slightly from 254 to 255 ns. Residual feedback therefore does not improve every timing metric and should be enabled only when the innovation, BDS reliability, selected observation source, and source-stability duration satisfy the gating conditions defined in Section 4.7.

A full-record Allan-deviation calculation would mix stable operation with BDS degradation, the controlled outage, and source-switching transients. Because these processes are nonstationary, such a calculation could incorrectly attribute switching discontinuities to intrinsic frequency instability. The Allan-deviation analysis was therefore performed separately over stable BDS-available intervals and over the steady interior of the controlled BDS outage. For the BDS-available case, the intervals from 1 to 5 h, 9 to 11 h, and 17 to 23 h were evaluated independently. For the outage case, only the interval from 12.25 to 15.75 h was used, thereby excluding the switching boundaries at the beginning and end of the outage. For each averaging time, the second-difference contributions from the disjoint stable intervals were computed separately and pooled according to the number of valid samples; no differences were formed across disconnected interval boundaries.

**Figure 9:**
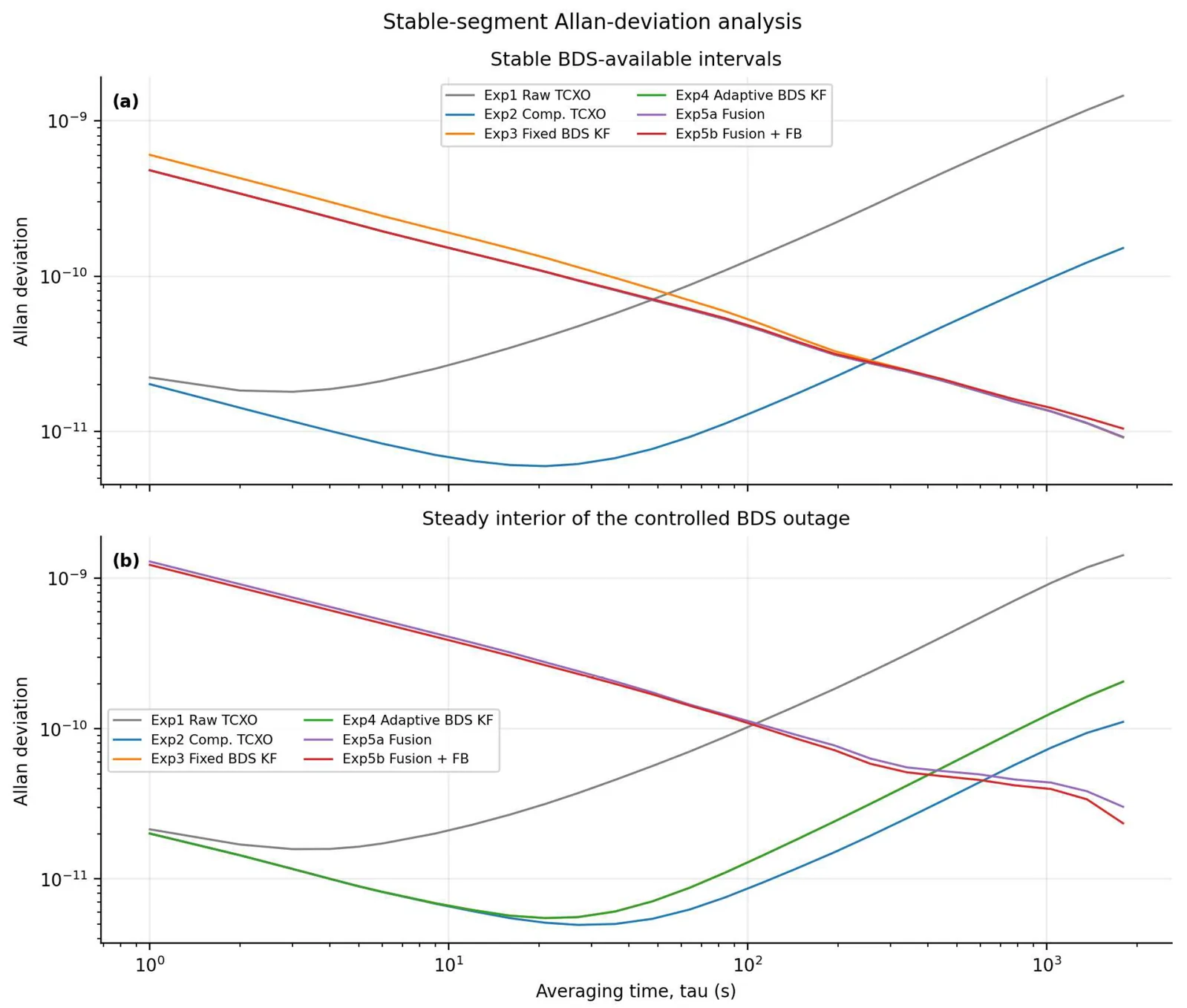
Stable-segment Allan-deviation analysis of the six methods: (**a**) stable BDS-available intervals of 1–5, 9–11, and 17–23 h; (**b**) the steady interior of the controlled BDS outage from 12.25 to 15.75 h.

As shown in Figure 9a, the digitally temperature-compensated TCXO exhibits the lowest Allan deviation at short averaging times because its output is not directly affected by external observation noise or measurement-update transients. The BDS-constrained and three-layer fusion methods exhibit higher short-term deviations because their output sequences include BDS observation noise and Kalman measurement corrections. As the averaging time increases, however, the Allan deviations of the externally constrained methods decrease, whereas those of the raw and compensated TCXOs increase because of residual frequency drift, oscillator aging, and low-frequency noise. The results demonstrate the complementary roles of the TCXO and the external observations: the local oscillator provides superior short-term smoothness, while the external timing sources constrain longer-term drift.

Figure 9b presents the corresponding analysis over the steady interior of the controlled BDS outage. During this interval, Exp3 and Exp4 receive no BDS corrections and operate primarily through state prediction. Their Allan deviations consequently increase at longer averaging times as the prediction error accumulates. Exp5a and Exp5b continue to receive eLoran updates and therefore exhibit larger Allan deviations at short averaging times because of the lower eLoran update rate and its larger instantaneous residuals. At longer averaging times, however, their Allan deviations become lower than those of the prediction-only methods because the eLoran observations constrain the accumulated TCXO drift.

These results show that a larger short-term eLoran innovation does not necessarily imply an absence of long-term timing value. eLoran may reduce long-term drift while simultaneously increasing short-term output variability. Accordingly, the stable-segment Allan-deviation results should be interpreted together with the time-domain error, outage-error, and switching-jump metrics rather than as an independent measure of overall performance across nonstationary operating conditions.

## 6. Conclusions

This study developed a three-layer adaptive Kalman filter-based time-fusion framework to coordinate the complementary characteristics of a local TCXO, BDS timing observations, and eLoran backup observations. The digitally temperature-compensated TCXO provides continuous short-term local timing, BDS serves as the primary external observation source when its reliability is sufficient, and eLoran maintains an independent external timing constraint when BDS is degraded or unavailable. The terms “high-rate,” “middle-rate,” and “low-rate” describe only the relative update intervals of these three layers within the proposed architecture.

The revised Kalman filter uses a five-state model comprising the local clock-phase error, the residual fractional-frequency offset of the temperature-compensated TCXO, its frequency-drift rate, and the equivalent slowly varying BDS and eLoran link biases. The temperature-compensation module and residual-feedback loop remain outside the Kalman-filter state vector, thereby avoiding duplicate representation of the residual TCXO frequency error. The BDS and eLoran observation errors are each separated into an equivalent slowly varying link-bias state and a short-term random residual. Only the short-term residual remaining after bias separation is conditionally approximated as zero-mean Gaussian under the nominal conditions considered in this study. This approximation is adopted as a working model for Kalman filtering and does not imply that the complete BDS or eLoran observation error is Gaussian under multipath, NLOS propagation, impulsive noise, or other degraded conditions.

The source-specific link biases are separated from the local clock state through their distinct stochastic models, prior covariance constraints, and source-dependent observation equations. Nevertheless, these bias states are not assumed to be independently observable at every epoch when only one external source is active. Their estimates should therefore be interpreted as constrained equivalent link-bias estimates rather than direct measurements of individual physical propagation errors. The remaining deviations between the simulated bias trajectories and their estimates also indicate that bias-state accuracy depends on prior calibration, process-noise selection, observation availability, and the duration for which each source remains active.

The representative 24-h controlled simulation showed that digital temperature compensation reduced the accumulated free-running TCXO time-phase error from approximately 447 μs to 139 μs, corresponding to a reduction of about 69%. Its overall RMSE decreased from approximately 286 μs to 79 μs, a reduction of about 72%. However, the compensated time error continued to accumulate because of residual frequency offsets, oscillator aging, and low-frequency noise. Digital temperature compensation alone therefore improves the local oscillator but does not provide an independent medium- or long-term timing constraint.

With BDS observations available, the fixed and adaptive BDS Kalman filters reduced the overall RMSE to approximately 2.5 μs. Their maximum absolute errors were approximately 14 and 13 μs, respectively. The reliability-based adaptive covariance reduced the influence of degraded BDS observations, but it could not provide an external correction during the controlled BDS outage. Consequently, both BDS-only filters relied on TCXO state prediction during that interval, and their BDS-outage maximum errors remained approximately 13–14 μs.

Under the adopted eLoran propagation-error and calibration settings, the two three-layer fusion methods maintained an external timing observation during the BDS outage. Exp5a, without residual feedback, achieved an overall RMSE of 68.9 ns and a maximum BDS-outage error of 254 ns. Exp5b, with gated residual feedback, achieved an RMSE of 63.7 ns and a maximum BDS-outage error of 255 ns. These results distinguish two related but different effects: eLoran preserves external timing observability during a BDS outage, and, under the particular controlled error model used here, it also substantially improves the simulated outage accuracy relative to TCXO-only prediction. The latter improvement is not universal, because the actual performance of eLoran depends strongly on transmitter synchronization, propagation-delay calibration, ASF correction, TOA estimation, and the temporal characteristics of the residual link bias.

Residual feedback should be regarded as a conditional enhancement rather than an always-beneficial component. Compared with Exp5a, the gated-feedback method reduced the overall RMSE by approximately 7.5% and reduced the maximum source-switch jump from 179 to 125 ns, corresponding to an improvement of about 30%. However, its maximum absolute error and BDS-outage maximum error increased slightly from 254 to 255 ns. Therefore, feedback activation must depend on the normalized innovation, BDS reliability, the selected observation source, and source-stability duration. During degraded observations, abnormal innovations, or source switching, the feedback gain should be reduced or suspended to prevent additional drift or discontinuities.

The Allan-deviation analysis was performed separately over stable BDS-available intervals and over the steady interior of the controlled BDS outage, rather than over the complete nonstationary record. During stable BDS availability, the compensated TCXO exhibited the best short-term stability, whereas the externally constrained methods showed improved behavior at longer averaging times. During the steady outage interval, the eLoran-supported fusion methods exhibited higher short-term Allan deviations because of the lower update rate and larger instantaneous observation residuals. At longer averaging times, however, the eLoran observations constrained the accumulated TCXO drift more effectively than prediction-only operation. These results show that short-term residual magnitude and long-term timing constraint represent different performance characteristics. They should not be interpreted using a single full-record Allan-deviation curve contaminated by degradation, outage, and source-switching boundaries.

The conclusions above are limited to the present controlled simulation. The actual RINEX file was used to extract BDS signal-quality indicators, which were resampled from 30 s to the 1 s filtering cycle, whereas the corresponding timing observations, TCXO errors, and eLoran observations were constructed through controlled models. The results therefore do not constitute validation of absolute timing accuracy relative to BDT or UTC, nor do they empirically verify the assumed residual probability distributions. Broad practical conclusions require long-term experiments using actual TCXO, BDS, and eLoran timing measurements referenced to an independent high-accuracy time scale.

Future work will focus on three aspects. First, long-duration experiments using actual timing equipment and independently referenced BDS and eLoran timing residuals will be conducted to evaluate the statistical distributions, temporal correlations, and identifiability of the clock and link-bias states. Second, time-varying eLoran propagation and ASF models will be introduced, together with robust innovation monitoring and non-Gaussian outlier-handling methods. Third, the feedback gain, smoothing coefficient, process-noise parameters, switching thresholds, and hysteresis width will be jointly optimized and implemented on an embedded platform to evaluate real-time computational cost, long-term numerical stability, and performance under repeated observation degradation and source switching.

## Data Availability

The raw data supporting the conclusions of this article will be made available by the authors on request.

## References

[1] Hein G.W. (2020). Status, Perspectives and Trends of Satellite Navigation. Satell. Navig..

[2] Yang S., Yi X., Dong R., Ren Q., Li X., Shuai T., Zhang J., Gong W. (2023). A Method for Autonomous Generation of High-Precision Time Scales for Navigation Constellations. Sensors.

[3] Son P.-W., Park S.G., Han Y., Seo K. (2020). eLoran: Resilient Positioning, Navigation, and Timing Infrastructure in Maritime Areas. IEEE Access.

[4] Lombardi M.A. (2022). Multi-Source Common-View Disciplined Clock: A Fail-Safe Clock for Critical Infrastructure Systems. J. Res. Natl. Inst. Stand. Technol..

[5] Groves P.D., Jiang Z. (2013). Height Aiding, C/N0 Weighting and Consistency Checking for GNSS NLOS and Multipath Mitigation in Urban Areas. J. Navig..

[6] Wang H., Deng Z., Feng B., Ma H., Xia Y. (2017). An Adaptive Kalman Filter Estimating Process Noise Covariance. Neurocomputing.

[7] Yin Z., Yang J., Ma Y., Wang S., Chai D., Cui H. (2023). A Robust Adaptive Extended Kalman Filter Based on an Improved Measurement Noise Covariance Matrix for the Monitoring and Isolation of Abnormal Disturbances in GNSS/INS Vehicle Navigation. Remote Sens..

[8] Fang T.H., Kim Y., Park S.G., Seo K., Park S.H. (2020). GPS and eLoran Integrated Navigation for Marine Applications Using Augmented Measurement Equation Based on Range Domain. Int. J. Control Autom. Syst..

[9] Riley W.J. (2008). Handbook of Frequency Stability Analysis.

[10] Yang M., Yan B., Yang C., Jiang X., Li S. (2025). Research on the eLoran/GNSS Combined Positioning Algorithm and Altitude Optimization. Remote Sens..

[11] Institute of Electrical and Electronics Engineers (2023). IEEE Guide for Measurement of Environmental Sensitivities of Standard Frequency Generators.

[12] (2022). IEEE Standard Definitions of Physical Quantities for Fundamental Frequency and Time Metrology—Random Instabilities.

[13] Lin C.-Y., Huang Y.-W., Lin T.-H. (2022). A Temperature-Compensated Crystal Oscillator with Piecewise Polynomial Varactor Compensation Achieving ±3.75-ppm Inaccuracy from −30 °C to 90 °C. IEEE Trans. Circuits Syst. II Express Briefs.

[14] Wang Z., Wu J. (2020). A Method to Increase the Frequency Stability of a TCXO by Compensating Thermal Hysteresis. Sensors.

[15] Chang H., Gao S., Guo Y., Gong X., Pan Z., Wang R., Zhang W., Zhou C., Gao Y., Lu X. (2026). A Novel Atmospheric-Delay Correction Technique for Enhancing Satellite–Ground Two-Way Time Synchronization. Meas. Sci. Technol..

[16] Tu R., Zhang P., Zhang R., Liu J., Lu X. (2018). Modeling and Assessment of Precise Time Transfer by Using BeiDou Navigation Satellite System Triple-Frequency Signals. Sensors.

[17] Berardo M., Lo Presti L. (2016). On the Use of a Signal Quality Index Applying at Tracking Stage Level to Assist the RAIM System of a GNSS Receiver. Sensors.

[18] Deng J., Wang H., Wei S., Zhang A. (2024). A Stochastic Model Based on Optimal Satellite Subset Selection Strategy for Smartphone Pseudorange Relative Positioning. Sensors.

[19] Swaszek P.F., Johnson G.W., Shalaev R., Wiggins M., Lo S.C., Hartnett R.J. An Investigation into the Temporal Correlation at the ASF Monitor Sites. Proceedings of the 36th Annual Technical Symposium of the International Loran Association.

[20] Lebekwe C.K., Zungeru A.M., Astin I. (2021). Meteorological Influence on eLoran Accuracy. IEEE Access.

[21] Boyce C.O.L., Lo S.C., Powell J.D., Enge P.K. Mitigating Atmospheric Noise for Loran. Proceedings of the 19th International Technical Meeting of the Satellite Division of the Institute of Navigation (ION GNSS 2006).

[22] Liu S., Guo W., Hua Y., Kou W. (2023). eLoran Propagation Delay Prediction Model Based on a BP Neural Network for a Complex Meteorological Environment. Sensors.

[23] Di J., Fu J., Xu B., Wu M., Liu L., Jin X. (2024). Timing Performance Testing and Regularity Analysis of eLoran System. Appl. Sci..

[24] Li Y., Hua Y., Yan B., Guo W. (2020). Research on the eLoran Differential Timing Method. Sensors.

[25] Luo Y., Li J., Yu C., Xu B., Li Y., Hsu L.-T., El-Sheimy N. (2019). Research on Time-Correlated Errors Using Allan Variance in a Kalman Filter Applicable to Vector-Tracking-Based GNSS Software-Defined Receiver for Autonomous Ground Vehicle Navigation. Remote Sens..

[26] Miao Z., Shen F., Xu D., He K., Tian C. (2015). Online Estimation of Allan Variance Coefficients Based on a Neural-Extended Kalman Filter. Sensors.

[27] Liu K., Guan X., Ren X., Wu J. (2024). Disciplining a Rubidium Atomic Clock Based on Adaptive Kalman Filter. Sensors.

[28] Liu W., Qi T., Hu Y., Fu S., Han B., Hsieh T.-H., Wang S. (2025). An Improved Adaptive Robust Extended Kalman Filter for Arctic Shipborne Tightly Coupled GNSS/INS Navigation. J. Mar. Sci. Eng..

[29] Woo R., Yang E.-J., Seo D.-W. (2019). A Fuzzy-Innovation-Based Adaptive Kalman Filter for Enhanced Vehicle Positioning in Dense Urban Environments. Sensors.

[30] Kalman R.E. (1960). A New Approach to Linear Filtering and Prediction Problems. J. Basic Eng..

[31] Bucy R.S., Joseph P.D. (1987). Filtering for Stochastic Processes with Applications to Guidance.

[32] Zanetti R., DeMars K.J. (2013). Joseph Formulation of Unscented and Quadrature Filters with Application to Consider States. J. Guid. Control Dyn..

[33] Carpenter J.R., D’Souza C.N. (2018). Navigation Filter Best Practices.

[34] Slock D.T.M. (1993). On the Convergence Behavior of the LMS and the Normalized LMS Algorithms. IEEE Trans. Signal Process..

[35] Peretic M., Gilabert R., Carroll J., Gutierrez J., Moore A., Christie J., Dill E.T. Statistical Analysis of GNSS Multipath Errors in Urban Canyons. Proceedings of the 2025 IEEE/ION Position, Location and Navigation Symposium (PLANS).

